# Data-driven load profiles and the dynamics of residential electricity consumption

**DOI:** 10.1038/s41467-022-31942-9

**Published:** 2022-08-06

**Authors:** Mehrnaz Anvari, Elisavet Proedrou, Benjamin Schäfer, Christian Beck, Holger Kantz, Marc Timme

**Affiliations:** 1 Potsdam Institute for Climate Impact Research (PIK), Member of the Leibniz Association, P.O. Box 60 12 03, D-14412 Potsdam, Germany; 2 DLR Institute for Networked Energy Systems, Oldenburg, Germany; 3 School of Mathematical Sciences, Queen Mary University of London, London, UK; 4 Faculty of Science and Technology, Norwegian University of Life Sciences, 1432 Ås, Norway; 5 Institute for Automation and Applied Informatics, Karlsruhe Institute for Technology, Karlsruhe, Germany; 6 The Alan Turing Institute, London, UK; 7 Max Planck Institute for the Physics of Complex Systems, D-01187 Dresden, Germany; 8 Chair for Network Dynamics, Center for Advancing Electronics Dresden (cfaed) and Institute for Theoretical Physics, Technical University of Dresden, 01062 Dresden, Germany; 9 Lakeside Labs, 9020 Klagenfurt am Wörthersee, Austria

**Keywords:** Applied physics, Nonlinear phenomena, Energy science and technology

## Abstract

The dynamics of power consumption constitutes an essential building block for planning and operating sustainable energy systems. Whereas variations in the dynamics of renewable energy generation are reasonably well studied, a deeper understanding of the variations in consumption dynamics is still missing. Here, we analyse highly resolved residential electricity consumption data of Austrian, German and UK households and propose a generally applicable data-driven load model. Specifically, we disentangle the average demand profiles from the demand fluctuations based purely on time series data. We introduce a stochastic model to quantitatively capture the highly intermittent demand fluctuations. Thereby, we offer a better understanding of demand dynamics, in particular its fluctuations, and provide general tools for disentangling mean demand and fluctuations for any given system, going beyond the standard load profile (SLP). Our insights on the demand dynamics may support planning and operating future-compliant (micro) grids in maintaining supply-demand balance.

## Abstract (web summary)

In modern power grids, knowing the required electric power demand and its variations is necessary to balance demand and supply. The authors propose a data-driven approach to create high-resolution load profiles and characterize their fluctuations, based on recorded data of electricity consumption.

## Introduction

Electrical energy is an essential part of daily life and is continuously generated, transmitted, stored and, finally, consumed. Generation and storage of electricity shall match the dynamic consumption of residential, industrial and other sectors at all times. To maintain the balance between the electricity generated by energy providers, and the electricity consumed by consumers, energy suppliers need to estimate the electricity required by all consumer sectors on a broad range of time scales from seconds to days. Estimating the typical variations in the electricity demand over the course of a day yields a load profile, which can be attained either through the definition of a methodology to extract a load profile from empirical data, the creation of a model or a combination of both.

Research aiming at developing load profiles goes back to at least the 1940s^1^, however, the issue of finding a precise, high-resolution load profile is becoming more and more urgent due to the increase of population, electrical heating systems, electrical vehicles for transportation, solar home systems as well as the increasing share of fluctuating renewable energy (RE) feed-in and the construction of distributed power grids, especially smart grids. In 1999, the first methodologically systematic German household load profile, known as the H0 Standard Load Profile (H0 SLP), was developed^2^ and has since been in use without alterations in at least Germany and Austria^3^, see also Supplementary Note 6 for translations of these German language references.

We focus here on the residential sector consuming around 29% of all electricity in the European Union^4^, and it is expected that its electricity consumption will considerably increase because of the electrification of both the transport and the heating system. Although initially household load profiles used a temporal resolution of around one hour, newer models have a temporal resolution up to and including one second, due to the recent availability of highly resolved datasets of electricity consumption and the usage of smart metres in selected houses, see Supplementary Note 1. These new datasets allow the grid operators to record the electricity consumption of individual houses at high temporal resolutions. Several previous studies^5–7^ analysed high temporal resolution datasets and reported the presence of extreme and significant peaks in the loads which have not been reported for datasets with temporal resolutions of 15 min to 1 h. However, a structured approach to translate these high-resolution datasets into usable load profiles is not readily available to date. Therefore, in this work, we first analyse highly resolved electricity consumption data for groups of houses in Austria, Germany and the UK. Our data-driven analysis indicates the potential for the presence of strong fluctuations and high levels of unpredictability in the distribution grids, see “Complex demand dynamics—the necessity of new load profiles”. Based on this analysis, we introduce a load model where we disentangle an average load profile from the fluctuations on top of the baseline. We thereby obtain a new load profile that is consistent with high-resolution electricity consumption data.

As mentioned above, one important reason of having a high-resolution load profile is the increasing share of RE feed-in. In contrast to electric energy generated from burning fossil fuels, RE feed-in is weather-dependent, intermittent, and highly variable^8–10^. It thus becomes harder to balance supply and demand. As the share of renewable feed-in is increasing, recent and current research focuses on gaining a deeper understanding of the electricity generated by RE as well as advanced approaches of balancing demand and supply, e.g., by load-shifting^11,12^. In contrast, the dynamics of electricity consumption is far from understood, in particular on the residential level, partially because electricity consumption data are difficult to obtain.

In this article, we offer a method for (a) extracting a new type of load model from a given set of consumption data to disentangle the baseline demand dynamics over the course of a day (average load profile, ALP) and the fluctuations on top of that baseline (stochastic fluctuation profile, SFP) and (b) better understanding the consumer demand dynamics. Taking into account available microscopic data, the model is capable of characterising (i) the demand of already moderately sized sets of consumers with (ii) high temporal resolution and (iii) applicable to a range of data, including those recorded in the future (and not restricted to those analysed in this article). Together, these features and capabilities go far beyond what the standard load profile (H0 SLP) offers and our approach is distinct from more recent complementary approaches since our model does not require microscopic parameters for consumer behaviour, consumer appliances, house infrastructures or other features that other models depend on.

After reviewing the need for a new demand model in more technical detail (see the section “Complex demand dynamics—the necessity of new load profiles”), we analyse the electricity consumption data of Germany, Austrian and UK houses measured for several weeks. We disentangle the variations in the consumption dynamics into two main factors, the average load profile (ALP) and the statistics of short-time fluctuations of the ALP. First, through the application of the empirical mode decomposition (EMD)^13^, we extract the average load profile from the time series data. This extracted trend captures the demand much more accurately than the often-used H0 SLP (see the section “Demand trend: mode decomposition”). Next, we use superstatistics to model the fluctuations around this trend into a stochastic fluctuation profile (SFP). Combining the trend and fluctuation analysis, we successfully reproduce a synthetic high-resolution load profile, yielding a full data-driven load profile (DLP). Our modelling approach readily transfers to use on other datasets. To facilitate such a transfer, we are providing executable code (see the section “Demand fluctuations: stochastic model”). We thereby develop a load profile methodology that is applicable to existing power grids and datasets, and also provide the tools for extracting load profiles in different regions and under different boundary conditions, for instance for microgrids with high shares of on-site generation or electric cars.

## Results

### Complex demand dynamics—the necessity for new load profiles

Notwithstanding recent advances, energy suppliers still mostly use the older load profiles, such as the H0 standard load profile, which only has a 15-min temporal resolution. In Germany, the standard load profile (H0 SLP), was introduced in 1999. Ninety percent of the residential load data used in its creation were measured in the 1970s or earlier with an hourly temporal resolution. Only 10% of the measured load data had a temporal resolution of 15 min. Here, we review some of the recent advances towards a modern load profile and touch on the persistent need for a generally applicable consumption framework which we provide in the next sections.

Three well-known model classes exist to describe a household load profile. Top-down or conditional demand analysis models are downward models that use the total electricity consumption estimates of multiple households as well as macro-variables to model the dynamics of household energy consumption by generating household load profiles^14,15^. Bottom-up models use micro-variables as input, such as the number of active occupants, the appliances’ energy demand and usage time etc. They also often use Markov chains to generate household load profiles^16,17^. Finally, hybrid models employ a combination of the techniques used in top-down and bottom-up models to build up a Statistical Adjusted Engineering (SAE) model^18^. A detailed analysis of how these models have been applied was recently reviewed in ref. ^19^. At present, many demand analysis models exist that can generate daily residential electricity load profiles, see Supplementary Note 1,^1,20^ for details. Only a few of these models use a high temporal resolution of the order of seconds and those require a lot of micro-parameters, which still leaves us with the need for an accurate, high-resolution, easy-to-use load profile to be developed.

**Fig. 1:**
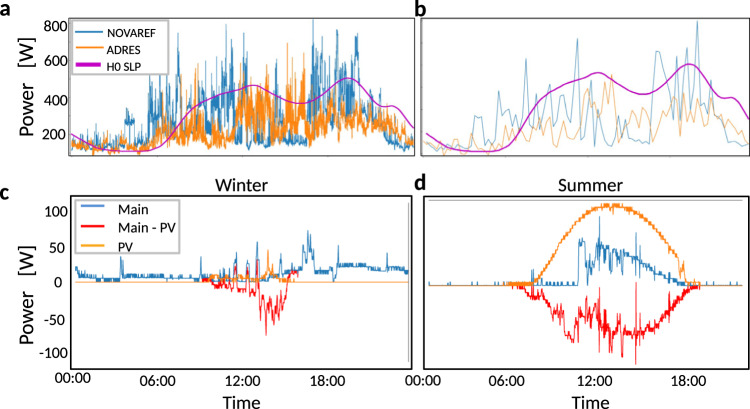
Systematic, asymmetric and intermittent deviations of empirical power consumption dynamics and German standard load profile (H0 SLP). **a** We compare the H0 SLP with the averaged real consumption data of a single day in winter for the NOVAREF project, the German dataset recorded between 2013 and 2016 including 12 houses, and ADRES project, the Austrian dataset recorded between 2009 and 2010, including 30 houses. The averaged real consumption data are created by taking the recorded electricity consumption of all houses in the measured time interval and, then, evaluating ${P}_{avg}(t)=\frac{1}{N}\mathop{\sum }\nolimits_{i}^{N}{P}_{{h}_{i}}(t)$, where ${P}_{{h}_{i}}$ is the measured electricity consumption of each house and *N* is the number of houses, which is respectively 12 and 30 for the NOVAREF and ADRES data. All three datasets were upsampled to a sampling rate of 2 s (0.5 Hz). The H0 SLP not only fails to capture the correct daily trend, but we also observe large fluctuations of the consumption at short time scales. **b** Shows the same datasets at 15-min resolution to emulate the temporal resolution that was used to generate the H0 SLP. Here the failure of the H0 SLP to capture the correct daily trend is even more pronounced. **c**, **d** show respectively the recorded household power consumption in winter (January 1, 2017) and summer (June 30, 2017) with and without photovoltaics as well as the electricity generated by the PV module installed on the roof of a house at a sampling rate of one minute (PV data source:^70^). Note the smaller spikes visible in panels (**a**, **b**) because the data is averaged over 12 and 30 houses, respectively, for NOVAREF and ADRES, while in panel **c** and **d** only a single household is shown.

A focus on the higher temporal resolution is necessary to fully understand modern consumption patterns and respond quickly, for instance the disturbances caused by input fluctuations or regulatory or trading anomalies^21^. Recent statistical research on power consumption (see for example, refs. ^6^ and ^7^) demonstrates that substantial differences exist between the statistical features of the highly resolved power consumption and consumption on a 15-min time scale. Analysing power consumption on the short time scale of seconds to one minute reveals extreme consumption spikes, which are completely ignored in the 15 min load profile^6^. In Fig. 1a, b, a comparison of the H0 SLP with the load profiles of two residential datasets of high temporal resolution, measured in Germany and Austria is shown. The Austrian dataset was recorded in 2009–2010 during the ADRES project^22^. The German dataset was recorded between 2013 and 2016 during the NOVAREF project^23^. In Supplementary Note 2, we report further on analysis datasets related to 70 households, recorded in August and September 2019 in Germany during the ENERA project.

As can be seen in Fig. 1a, b, the averaged single-day load profiles of both the ADRES and the NOVAREF datasets, strongly deviate from the H0 SLP. Specifically, the trend (mean) of the load profile of the measured data is not well described by the H0 SLP and the fluctuations on short times scales are significant (see “Demand trend: mode decomposition”). Furthermore, the analysis of the statistics of the highly resolved power consumption reveals the presence of non-Gaussian, intermittent and asymmetric fluctuations, which need to be taken into account when designing demand side management and control mechanisms, see “Demand fluctuations: stochastic model” and Supplementary Note 4.

As Fig. 1a, b illustrates, the H0 SLP data clearly deviates from the consumption pattern indicated by the NOVAREF and ADRES data. There are three main reasons for that. First, due missing input of microscopic data, the H0 SLP is hardly capable of accurately capturing the electricity consumption. Second, the H0 SLP is based on data that were measured mostly in the 1960s and 1970s with a small fraction measured towards the end of the 1990s. Since then electricity consumption behaviours and devices have changed drastically. Third, moderate numbers of consumers (e.g., a group of 12 houses considered in this work) do not typically align with an overall average for a larger group of consumers (that might produce a result whose consumption pattern might be closer to the H0 SLP).

Some of the observed stochastic properties could be explained by the increased usage of new power generation and new consumption devices, for instance rooftop photovoltaic panels (PVs) and electronic devices such as electrical heating systems, smartphones, tablets, robot vacuum cleaners, electric vehicles etc.^24^. The usage of such devices will influence and alter the load dynamics and in particular its time-dependent average—the load profile. As shown in Fig. 1c, d, the consumption can even reach negative values if houses are directly connected to local RE resources^25^. However, these negative values are less or even reach zero during the winter time because of the reduction of the PV, or the usage of electrical heating. Local fluctuations in solar and wind generation may further lead to coincident load profile spikes, both positive and negative. Therefore, precisely analysing and modelling the power consumption of households on a short time scale is essential to ensure the stability of distributed grids. In addition to installing more on-site generations in the residential sector, replacing fossil-fuelled cars with electric ones, which are charged using household electricity, results in changes in the shape of the load profile of the households, especially when organising consumers in microgrids^26^, see also Supplementary Note 2. Since we expect an increasing number of such electrical cars, as well as an increasing penetration of strongly fluctuating RE generating electricity, a new data-driven load profile based on modern and highly resolved measurements is urgently needed.

As mentioned above, extreme consumption spikes are completely ignored in the 15 min load profile as they are averaged out. However, these spikes are of particular importance to lower voltage distribution grids, where coincident consumption can dominate the consumption patterns locally or on a country scale, due to e.g., synchronised activity during major (e.g., sports) events, such as TV pickup in Britain^27^ as well as German and U.S. power system frequency variation, respectively, during the 2010 World Cup semi-final game and Super Bowl^28,29^. A well-known simple measure to quantify the coincident electricity consumption between households and moreover to see how this coincidence depends on the number of households is the diversity factor between households^30^. To determine the diversity factor between households, we first evaluate the maximum coincident demand, *P*_*cd*_ and non-coincident demand, *P*_*ncd*_, respectively in 15 min and 1-day time windows. For this purpose, we sum the maximum power demand of all houses every 15 min and then divide the *P*_*cd*_ with the sum of the maximum power demand of all houses over the course of a day, i.e., *P*_*ncd*_.

**Fig. 2:**
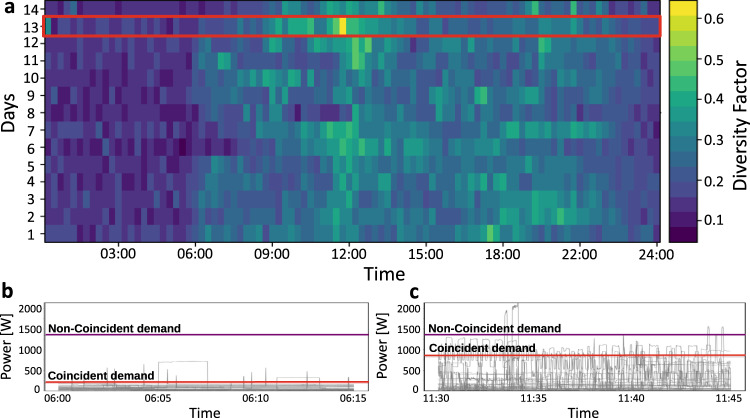
Indicating the interaction between households' energy consumption by considering the diversity factor. **a** shows the diversity factor or the ratio between coincident demand, *P*_*cd*_, and non-coincident demand, *P*_*ncd*_, of 30 households belonging to the ADRES dataset every 15 min and for 14 days. As it is clear, there are some time intervals during a day whose diversity factor reaches 0.6. **b**, **c** respectively show the energy consumption trajectory of all 30 households from 6:00 to 6:15 and 11:30 to 11:45. It is clear from the figures that the energy consumption of all houses is low around 6:00 and then is gradually increasing together around 11:00 and, consequently the difference between *P*_*cd*_ and *P*_*ncd*_ is decreasing proving the obvious interaction between houses consuming the electricity.

The diversity factor varies from zero to one, where zero indicates no coincident electricity consumption between households, while a diversity factor equal to one shows strong coincidence. As an example, the diversity factor of the ADRES dataset, sampled every 15 min for 14 days, is shown Fig. 2a. To clearly indicate the interaction between houses during a day, (i) the energy consumption trajectory of all 30 households (in grey); (ii) the maximum coincident demand (in red) and (iii) the non-coincident demand (in purple) between 6:00 to 6:15 (Fig. 2b) and 11:30 to 11:45 (Fig. 2c) for day 13 are shown in Fig. 2b, c.

Looking at the households trajectories, it is clear that the energy consumption of all houses is low at the beginning of the day and then it is gradually increasing until around 11:00. Thus, the value of the diversity factor, which is the ratio between coincident demand (*P*_*cd*_), and the non-coincident demand (*P*_*ncd*_) increases during daytime. This proves the interaction between the demand of the households during the day and is the reason why the significant spikes in average energy consumption do not disappear after averaging.

To show the relationship between the value of the diversity factor and the number of households, we calculate the diversity factor for the NOVAREF and ENERA datasets, which are composed of 12 and 70 households, respectively, in Supplementary Note 2. Our results demonstrate that the values of the diversity factor are not zero during a day, even for the 70 ENERA houses and for some time intervals the diversity factor ranges between 0.3 and 0.4 or even larger (maximum 0.6). This indicates that regardless of the number of houses coincident demand will take place during certain time intervals (e.g., lunch and dinner time) and spikes during that time will not be averaged out. This will result in a spiky load profile, such as the single-day averaged ENERA load profile, see also Supplementary Note 1.

In the next sections, we introduce a generally applicable methodology for creating residential load profiles and demonstrate its applicability through comparisons with power consumption datasets measured in Germany and Austria, as well as the industry standard load profile. In the next section (“Demand trend: mode decomposition”), we present a methodology to extract the averaged load profile (ALP).

### Demand trend: mode decomposition

In this section, we present a methodology with which the Averaged Load Profile (ALP) from high temporal resolution datasets is extracted, and the load profile of a full week is created. The latter explicitly takes into consideration not only the differences existing between workdays and weekends as much as the H0 SLP does, but also the differences between different weekdays unlike the H0 SLP. The main advantage of the ALP compared to the H0 SLP is that it only requires a few weeks of high-resolution measured data and can, therefore, be applied to both small neighbourhoods and whole cities. Furthermore, its only input is the electricity consumption of the houses, unlike most existing models, which reply on numerous micro- or macroscopic parameters. As a result, it can easily be used to analyse both present and future residential power systems, which might include new technologies and devices (see also Supplementary Note 5 for applications on a different time of the year and a different dataset).

Due to the high temporal resolution of the datasets we use, the extracted ALP has a higher level of accuracy than the H0 SLP. It is, therefore, a good alternative to be used instead of the H0 SLP and could provide better and more accurate results, especially when investigating or operating microgrids (a detailed discussion on the subject can be found in “Discussion”). In its present form, the ALP can capture the weekly trend of the load profile of a group of houses (anywhere from 12 to 70 houses have been tested).

To produce the ALP, we first extract the consumption trend from four consecutive weeks (or seven consecutive weekdays or weekends) from high-resolution electricity consumption data measurements, as explained in Supplementary Notes 3 and 5.

In this section, we present our results for the NOVAREF dataset (temporal resolution = 2 s).

**Fig. 3:**
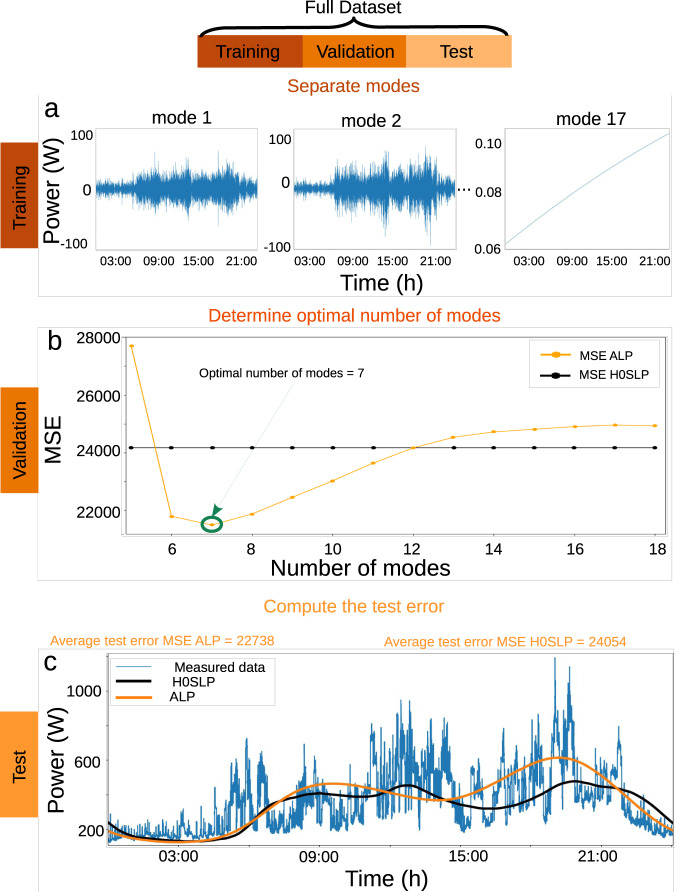
Training and validation are used to compute the optimal number of modes, avoiding over- and underfitting. The full dataset is split into training, validation and test datasets (shown at the top of the Figure). **a** Using the EMD, we decompose the training set into its empirical modes. **b** Next, the validation set is used to optimise the number of modes, avoiding both under- and overfitting. Including too many modes overfits the data but yields poor results on the test datasets. Vice versa, a model with too few modes underfits the data and fails to capture its main trend. Hence, we systematically determine the optimal number of modes by minimising the mean-squared error (MSE). **c** Finally, using the *N*_*optimal*_, we extract ALP for the test set. Computing the MSE of the ALP obtained for each week demonstrates that the ALP performs better than the H0 SLP, since it tracks the measured energy consumption more closely compared to the H0 SLP. As shown in panel (**c**), the average value of the MSE ALP for all weeks is lower than that of the MSE H0 SLP. For the purpose of clarity only a single day is shown here (28.05), see also Supplementary Note 5.

The methodology we use to determine the ALP is the following, and it is illustrated in Fig. 3. Using the Empirical Mode Decomposition (EMD)^13^, we split the dataset into multiple modes and, thereby, separate the long-term trends (high-number modes) from the short-term fluctuations (low-number modes). Since the EMD extracts all signals present in the data, the original dataset can be recreated 1-to-1 without any loss of information by summing up all the modes. Next, the data is split into training, validation and test datasets. The training data is used to generate the load trend by summing a certain number of long-term trend modes. During the validation step, the optimal required number of modes is determined to avoid both under- and overfitting. Finally, the extracted ALP is used to compute a test error from the test set, using the optimal number of modes obtained from the previous step. A detailed discussion of the adaptive time-frequency data analysis via the EMD can be found in “Methods”, Supplementary Note 3 and in a recent paper applying EMD in a stochastic context^31^.

Before training, we apply the EMD method on the entire dataset and, thereby, extract 17 individual and independent modes. We then divide these modes into two categories: (a) high-frequency; and (b) low-frequency modes. The high-frequency modes contain information on the stochastic consumption fluctuations (see “Demand fluctuations: stochastic model”), while the low-frequency modes are almost free of stochastic fluctuations and are instead dominated by deterministic effects. To begin the training step, we first choose 4 chronologically consecutive weeks of the measured NOVAREF dataset containing no data gaps, i.e., 01.07–01.14, 01.14–01.21, 02.04–02.11, 02.11–02.18, which were recorded during the winter time between 2013 and 2016. These 4 weeks are the training set. Then, we calculate the average of these four weekly consumption data and, correspondingly, we average the last *N* low-frequency modes of these weeks.

In order to get the best performing ALP, we must determine the optimal number of modes *N*, i.e., *N*_*optimal*_, from the validation set. If we sum too few modes, the extracted ALP fails to track the high-resolution consumption better than the H0 SLP. In contrast, if we sum too many modes it will overfit the dataset and lead later to an inaccurate consumption trend for the test set. To determine the *N*_*optimal*_ and quantify the performance of the optimised ALP, we use the mean-squared error (MSE) on the validation set, which consists of 9 randomly chosen weeks of the data measured during winter time (see “Methods” for details on MSE and the Supplementary Note 5 for a discussion of a different error metric, i.e., MAE). We must stress here that we used 9 weeks of data because these were the data that were available to us. For the purpose of applying this method on other, shorter, datasets only one additional week of data sound suffice to determine the optimum ALP.

The results of the above analysis determine that *N*_*optimal*_ ≈ 6. . . 8 for the NOVAREF validation dataset (see the optimal result in Fig. 3b). As seen in Fig. 3b, even for values of 8 < *N* < 12, the generated ALP outperforms the H0 SLP in terms of demand prediction. Consequently, the ALP determines the daily and weekly electricity consumption trend of the 12 NOVAREF households much more accurately compared to the H0 SLP. However, as mentioned before, for *N* > *N*_*optimal*_, the ALP overfits the data, and hence later gives an inaccurate results for the test set.

Finally, we use 4 randomly chosen winter weeks to test the extracted ALP. We find that the ALP outperforms the H0 SLP when the *N*_*optimal*_ is chosen, as is evident from its lower average value of MSE, which is reported in the text above the graph in Fig. 3c. It should be noted that the ALP does not perform as well for the smaller or larger number of modes. Figure 3c shows the results for a single day of a week of the test set. We continue the evaluation of the ALP performance in the next section, i.e., “Demand fluctuations: stochastic model”.

Here, we focus on the analysis of the NOVAREF dataset. We determined the optimum ALP for the winter weeks by training it and determining the related *N*_*optimal*_. In addition, we investigate the seasonality of the data in more detail in Supplementary Note 5. In particular, we demonstrate that the optimum ALP for the summer weeks still follows the demand trend visible in the winter weeks, and outperforms the H0 SLP in the winter season. We show also the applicability of our methodology by applying it on a UK household dataset in Supplementary Note 5.

The hereby introduced ALP has several major advantages compared to both the H0 SLP and the available demand models. Firstly, it is data-driven and is based on modern, high temporal resolution (2 s) measurements, whereas the H0 SLP is based mostly on hourly resolution data measured before 1999, with a small subset of 15-min resolution data measured between 1990 and 1999^2^. Secondly, because only four chronologically consecutive weeks of data are necessary to generate the weekly ALP, and only a few additional weeks of data are necessary to determine and validate the *N*_*optimal*_, it can be used to extract the averaged load profile of both small and large groups of houses and does not need the plethora of the micro-and-macro-parameters that presently available demand models require. Note that here, we introduce a methodology with which the baseline demand dynamics over the course of a day can be determined from a measured electricity consumption. In “Demand fluctuations: stochastic model”, a stochastic model to generate the stochastic fluctuations on top of this baseline is presented.

### Demand fluctuations: stochastic model

Having extracted the predominantly deterministic trend of consumption, we now turn to the stochastic fluctuations around this trend. Specifically, we split the power consumption into trend *P*^(trend)^ and fluctuations *P*^(fluc.)^:

1 $$P={P}^{{{\rm{(trend)}}}}\left(t\right)+{P}^{{{\rm{(fluc.)}}}}\left(t\right).$$

**Fig. 4:**
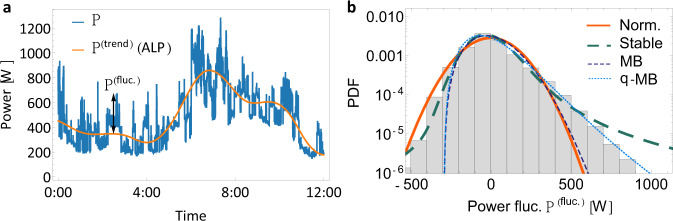
The intermittent characteristic of power consumption fluctuations. **a** The total power consumption *P* is a sum of the trend consumption *P*^trend^, obtained by the EMD method described in the previous section and fluctuations *P*^fluc.^. We record the difference between trend and real demand as the fluctuation trajectory. For the purpose of clarity only a single day is shown here. **b** The probability density function (PDF) of the consumption fluctuation does not follow a Gaussian distribution but is better described by a *q*-Maxwell–Boltzmann distribution, especially on the right flank. The histogram uses the whole NOVAREF dataset. Alongside the Gaussian, we show a stable and Maxwell–Boltzmann (MB) distribution. The *q*-Maxwell–Boltzmann parameters are determined by the methods of moments. See also Supplementary Note 4 for further details.

First, we investigate the statistical properties of these consumption fluctuations. Analysing the histograms, we notice that the fluctuations are skewed, i.e., asymmetric, and heavy-tailed, so large deviations are much more likely than if they were characterised by a simple Gaussian distribution, see Fig. 4.

**Fig. 5:**
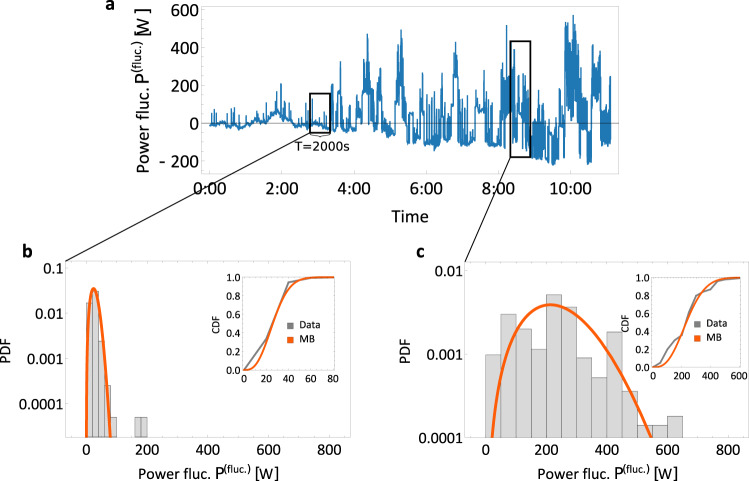
Local Maxwell–Boltzmann distributions of power demand fluctuations. **a** Using only the high-frequency modes from the empirical mode decomposition (EMD), we plot the consumption fluctuations *P*^(fluct.)^ over time. **b**, **c** Using superstatistical methods^32, 33^, we find a long-time scale *T* ≈ 2000 s, see also Supplementary Note 4. On this local time scale *T*, we observe local Maxwell–Boltzmann distributions, which can be very narrow (**b**) or broad (**c**). The histograms contain only 1000 (length of *T*, given a 2 s resolution) data points of *P*^(fluct.)^ as local snapshots and we plot MB fits as references. In addition to the PDF, we compare the cumulative probability functions (CDF) of MB distribution with the data in the inset. All plots use the NOVAREF data from a test set in April. The time period and day are chosen for illustration purposes only.

Next, we construct a stochastic model, which describes the observed consumption fluctuation statistics by applying superstatistics methods^21,32–35^: Dividing the full trajectory into several shorter trajectories allows us to characterise each local distribution with a simpler distribution, such as a Gaussian or an exponential distribution. In the case of consumption fluctuations, the fluctuations within each time window of approximately *T* ≈ 2000 s follow a distinct Maxwell–Boltzmann distribution, see Fig. 5. See Supplementary Note 4 for details on the superstatistical procedure, as well as the evaluation of this long-time scale *T* (see also Supplementary Fig. 13). Each local Maxwell–Boltzmann distribution has its distinct scale parameter *σ*_*MB*_ and offset from zero *μ*_*MB*_. When we now move our analysis from one local distribution to the next one, we observe a slow time dynamics of these Maxwell–Boltzmann parameters *σ*_*MB*_ and *μ*_*MB*_ and thereby a time dynamics of the local Maxwell–Boltzmann distribution itself. Superimposing these time-varying local distributions, we re-obtain the full aggregated statistics as approximately a q-Maxwell–Boltzmann distribution, see Fig. 4.

Mathematically, we formulate stochastic equations of motion for the fluctuations leading to local Maxwell–Boltzmann distributions as follows: We define auxiliary variables *x*_*i*_, with *i* ∈ {1, 2, . . . , *J*}, each following a simple Ornstein-Uhlenbeck process, based on independent Wiener processes *W*_*i*_:

2 $${{{{{{{\rm{d}}}}}}}}{x}_{i}\left(t\right)=-\gamma {x}_{i}\left(t\right){{{{{{{\rm{d}}}}}}}}t+\epsilon {{{{{{{\rm{d}}}}}}}}{W}_{i},$$

with damping *γ* and fluctuations amplitude *ϵ*. Hence, the *x*_*i*_ are identical but independently distributed Gaussian random variables with a mean 0 and standard deviation $\sigma =\frac{\epsilon }{\sqrt{2\gamma }}$. Then, the demand fluctuations *P*^(fluc.)^ are obtained by aggregating these Gaussian distributions and applying the observed shift from zero *μ*_*MB*_:

3 $${P}^{{{{{{{{\rm{(fluc.)}}}}}}}}}\left(t\right)=\sqrt{{\left({x}_{1}(t)\right)}^{2}+{\left({x}_{2}(t)\right)}^{2}+...+{\left({x}_{J}(t)\right)}^{2}}+{\mu }_{MB}.$$

As is known from statistical physics^36^, choosing *J* = 3 yields exactly Maxwell–Boltzmann distributions in the probability density *p*:

4 $$p\left({P}^{{{{{{{{\rm{(fluc.)}}}}}}}}}\right)=\frac{1}{{\sigma }_{MB}^{3}}\sqrt{\frac{2}{\pi }}{\left({P}^{{{{{{{{\rm{(fluc.)}}}}}}}}}-{\mu }_{MB}\right)}^{2}\exp \left[-\frac{{\left({P}^{{{{{{{{\rm{(fluc.)}}}}}}}}}-{\mu }_{MB}\right)}^{2}}{2{\sigma }_{MB}^{2}}\right],$$

where the shape parameter *σ*_*MB*_ of the local Maxwell–Boltzmann distribution is identical to the standard deviation of the independent Gaussian variables *σ*_*MB*_ = *σ*. We do consider cases of *J* ≠ 3 in Supplementary Note 4 and find that *J* = 3 is the best fit to the data. Indeed, the approach of three independent Gaussian variables is very convenient for computational application. Alternative modelling approaches using a mathematically simple 1-D process would require more complex dynamics.

In applying the superstatistical approach, we implicitly assumed separation of time scales. Here, we have the long-time scale, on which we locally observe Maxwell–Boltzmann distributions of the power fluctuations of *T* ≈ 2000 s (Fig. 5). Furthermore, we estimate the short time scale, on which each local distribution relaxes to its equilibrium, based on the autocorrelation decay of the data as *τ* = 1/*γ* ≈ 300. . . 400 s, see also Supplementary Note 4. Comparing these two time scales, we observe a clear time separation between long-time scales *T* and short time scales *τ*, which differ by a factor of 20. Hence, each local Maxwell–Boltzmann distribution relaxes much faster towards its equilibrium (with rate 1/*τ* = *γ*) than the overall process changes towards a new Maxwell–Boltzmann distribution (which happens with a rate of 1/*T*).

**Fig. 6:**
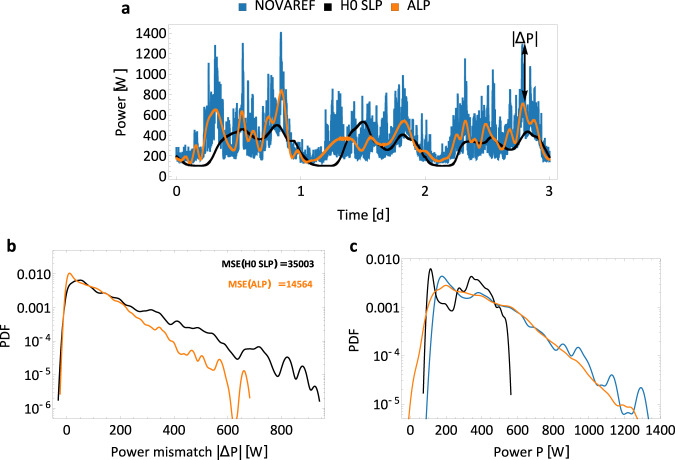
Synthetic power demand in agreement with empirical data. **a** We display a brief trajectory of the real (blue), H0 SLP (black) and the new ALP (orange). The ALP curve overall keeps closer to the real consumption values than the H0 SLP does. **b** The histogram of the power mismatch Δ*P* = ∣*P*(real) − *P*(model)∣ shows higher deviations of the demand for the H0 SLP compared to the ALP. We also report the mean-squared error (MSE) of the H0 SLP and the ALP. **c** Combining the trend extraction via EMD to obtain the ALP and the superstatistical model for demand fluctuations (DLP) approximates the real consumption histogram very well, in particular for large consumption values. All trajectories use the same time stamps, starting 15 min past midnight on 15th of April of the NOVAREF data from test set. The histograms return almost identical results for other weeks.

Finally, we combine the EMD-based trend of the demand with the stochastic fluctuation model, obtaining a data-driven load profile (DLP) and, then, compare it to the original NOVAREF consumption data. The model enables us to make some rough general predictions for the near future connected to these training weeks. Long-term forecasting is outside our scope as there is insufficient data available. We notice that while the precise trajectories are not identical (by construction), the stochastic properties align very well with drastically reduced error compared to the standard H0 SLP model (see Fig. 6). In “Methods”, we provide a link to the software and pseudo-code to generate these kinds of trajectories for other demand regions and datasets.

## Discussion

Summarising, we have shown that modern residential electricity load profiles strongly differ from the H0 standard load profile (H0 SLP), which is widely used in the industry. We set out to replace or supplement the existing standard load profile and obtained three main results: First, using the empirical mode decomposition (EMD), we develop a model to extract baseline demand dynamics (ALP) over the course of a day. Second, using superstatistics, we identify characteristics of short-time fluctuations around this baseline and, hence, we developed a simple stochastic fluctuation profile (SFP) to describe the non-Gaussian fluctuations of the demand around the trend. Finally, combining both trend and fluctuations yields a full data-driven load profile (DLP).

Hereby, the introduced model specifies a typical range of power consumption at a given time of a day and allows us to study the variation of probabilities of certain extreme power consumption events. Knowledge of the expected demand is critical for energy providers to calculate how much power is needed by each household within a given time period. Simultaneously, knowledge of how much the demand might fluctuate around this trend is also essential, to have sufficient balancing and backup power at hand. According to the Bundesverband der Energie- und Wasserwirtschaft e. V. (BDEW) (German Association of Energy and Water Industries)^2^, the electricity load of a household can be expected to deviate between 10 and 20% at any given time. Our fluctuation model provides a better quantitative estimate for these fluctuations to avoid an overestimation of the demand and too much usage of expensive quickly dispatchable generation^37^. On the other hand, we have to prevent an underestimation of the demand as this could easily lead to a collapse of the system.

Another strong point of our modelling approach is its flexibility and broad applicability: We do not present a fixed load profile but a methodology to extract trend (ALP) and fluctuations (SFP) for any present or future power system. This data-driven approach can be applied to different consumer groups, regions or even continents without requiring external or microscopic knowledge. Almost all alternative models require a lot of micro-parameters or fine-grain measurements to achieve the load profile, such as the Markovian model and Hidden Markov Model introduced respectively in refs. ^38^ and ^39^. It is worth to mention that our model is distinct from most machine learning models, as they: (i) Typically require huge training datasets and aim for pattern prediction of data outside the training set and moreover; (ii) do not contribute to the insights our approach offers, e.g., in terms of baseline vs fluctuations (and their relevant time scales). As such our approach is complementary to existing approaches employing machine learning. Maybe even more importantly, the extracted profiles can easily be updated based on recent developments in consumption, e.g., due to additional PV installation or adaptation of electrical cars. Similarly, new load profiles can be created for newly built micro or smart grids^40^ or, even existing grids, such as UK. Many existing grids suffer from network congestion and, therefore, either require network reinforcements or operators need to utilise the flexibility of domestic loads to relieve grid load. We demonstrate how our approach is applied to a different group of households located in UK in Supplementary Note 5.

The H0 SLP focused mainly on the time scale of 15 min and reports much smaller fluctuations than our new load profiles. How can we explain this? First, we note that the H0 SLP used older data, with a temporal resolution much lower than the 1 s of our data sources. More relevant is however the power system perspective: Generation and demand are scheduled for fixed time intervals, such as 15 min and all fluctuations and deviations within each interval are taken care by control mechanisms^41^. This view also emphasises the role of the high-voltage transmission grid where some demand fluctuations, which we observe on the distribution grid, might not occur. Our temporally highly resolved model and focus on the distribution grid become increasingly relevant: Conventional generators and their stabilising inertia are removed from the grid and renewable generators are often directly coupled with households. Hence, the balance of supply and demand has to be present also on the distribution level and on an increasingly fast time scale. Consistently, high-resolution datasets, such as the now-published NOVAREF data are becoming even more important. Any future model, forecast or simulation relies on updated and high-resolution data, even more so when applying data-driven approaches^42^ or machine learning^43^. If no such data are available, any prediction will have an increasing error. Both households and the increasing share of renewables introduce fluctuations and without updated highly resolved data, we would face increasing uncertainties on both the expected demand and generation. These uncertainties would likely affect energy prices as well, making them more volatile as predictions of the power balance become less accurate. Finally, we note that not only do both households and renewables introduce fluctuations in the distribution power grid but their fluctuations share similarities. In particular, the heavy tails of consumption fluctuations and its slowly decreasing power spectrum are also observed in renewable generation^10^.

Our model is also especially useful in the case of microgrids which are often powered either partially or fully by renewable resources combined with smart metres. One such renewable resource is photovoltaic panels, which can be installed on rooftops, gardens^44^, walls^45^, walkways^46^ and over roads^47^. Two more examples are small vertical wind turbines^48^ and bladeless wind turbines^49^, both of which can be mounted on any flat, sturdy surface. They are very well suited for use in residential areas due to their small size and low noise output, especially the newer designs such as Flower Tulips^50^ and the Vortex Bladeless Turbine^49^. These renewable energy sources can be utilised not only by households^51^ but also by farms, small and large businesses^52^ as well as industries^53^ since they only require a sturdy flat surface and can be used in a stand-alone more or in combination with one another^54,55^, and allow them to go partially or entirely off-grid.

In the past, small autonomous grids were comprised mainly of remote, usually rural villages or households powered by fossil fuel generators. In recent years, the concept has been reconfigured to help overcome the challenges of integrating intermittent renewable energy production with the main energy grid^56,57^ as well as provide electricity to populations in both remote, island^58^ and urban regions as well as business parks, industrial parks and resorts^59^ while replacing fossil fuels with renewable resources, in both developed^59^ and developing countries^60,61^. Such grids, however, always face the problem of matching the intermittent energy generation with the, as we have shown in “Complex demand dynamics—the necessity of new load profiles”, also the intermittent power consumption of the households that comprise them.

A task that is made harder by the fact that many microgrids are composed of less than the 332 households that the H0 SLP assumes. As shown in “Complex demand dynamics—the necessity of new load profiles” and “Demand trend: mode decomposition”, the consumption behaviour of a small number of houses deviates significantly from the H0 SLP and displays a strong stochastic component. Which technologies can be used to achieve a successful balancing between the generation and consumption^56,62^, which protection schemes^63^ and control systems can be used to ensure the stable and secure operation of these grids^64–66^ and even more scheduling and operation issues^57,67,68^ are all active areas of research.

Furthermore, our model could allow the administrators of an autonomous or semi-autonomous micro-grid equipped with smart metres to use the collected data to create a general rough prediction of the electricity consumption of the microgrids units in the following weeks and take the appropriate measures to ensure a continuous stable operation, e.g., by ensuring enough balancing power is available. At present, the H0 SLP, which does not differentiate between the electricity consumption of a handful of houses and a small city, makes this impossible. Because our approach is entirely dependent on data with no prior assumptions being made, it could theoretically be used to extract the averaged load profile not only of households but also of small or large-scale of industry or, even, full countries. The current lack of freely available data makes this a task for the future. For example, we wish to apply our model on consumption data of other regions, and compare it to the local equivalent of the H0 SLP. Regions like Canada, where a large number of smart metres are already installed, are particularly promising. While we focused here on household demand, the presented methods should also be applicable to extract the demand trend and fluctuations of industry consumption or combinations of very different consumers. Furthermore, the fluctuation modelling could be extended to include longer-term correlations or match the precise increments in the observed datasets. This would move the study of consumption fluctuations closer to the well-researched state of fluctuations in renewable generation. Given sufficient high-resolution data in the future, other data-driven methods, such as recurrent neural networks, weighted-nearest-neighbour predictors or multilayer perceptrons could be trained on such data. These methods typically require constant data input, while our ALP provides a semi-static prediction. All data used here were taken prior to the COVID-19 pandemic, during which the time people spent at home typically increased substantially. How demand patterns might have changed due to COVID-19 and how well our framework could still capture the dynamics remains an interesting question for future research.

## Methods

### EMD

The Empirical mode decomposition (EMD) is a well-known method used for nonlinear, non-stationary datasets. It decomposes the data into a finite number of intrinsic mode functions based on the local properties of the data. Therefore, this method is not restricted to linear or stationary time series, as is the case with other methods, such as Fourier spectral analysis. To determine the empirical mode functions, one applies the following steps on a dataset: (1) first the envelope of both local maxima and minima in a dataset are defined separately. For instance, all local maxima are connected by cubic spline lines for the upper envelope and then the same procedure is repeated for the lower envelope. Consequently, all data are confined between upper and lower envelopes. It is worth mentioning that in this method there is no need for the data to have zero crossings, therefore all values can be just positive or negative. (2) Defining the upper and lower envelopes, their mean value, *m*_1_ has to be calculated and then subtracted from the original data, i.e., *X*(*t*) − *m*_1_ = *h*_1_, where *X*(*t*) is the original data and *h*_1_ is called the first component. (3) In the next step, the first component, *h*_1_ is converted to the intrinsic mode function (IMF), i.e., *h*_1_ should have the same number of extrema and zero crossings plus the symmetry of upper and lower envelops around zero (for details, see ref. ^13^). After finding the IMF, *c*_1_, we subtract it from the original data: *X*(*t*) − *c*_1_ = *r*_1_. This *c*_1_ is the first mode and contains the shortest period of the original data *X*(*t*). (4) Steps (1)–(3) are repeated until *r*_*n*_ becomes a monotonic function, and it becomes impossible to define any envelop for that. If we sum up all IMFs and the residue, we reproduce again the original data, i.e.

5 $$X(t)=\mathop{\sum }\limits_{i=1}^{n}{c}_{i}+{r}_{n};$$

Consequently, we can obtain all the intrinsic oscillation modes of the dataset with the EMD method.

### Trend extraction

In this section, we present the adaptive time-frequency data analysis we used to determine the ALP. It is based on a normalised version of the one-step prediction mean-squared error (MSE)^69^ of an estimator.

In order to generate the ALP, we separate firstly four chronologically consecutive weeks of high-resolution electricity consumption dataset with no data gaps as the training set. In this case, weeks 01.07–01.14, 01.14–01.21, 02.04–02.11, 02.11–04.18 of the NOVAREF dataset, which were recorded between 2013 and 2016. For accurate and meaningful results we must use data from the same group of houses whose demand trend is to be determined. The first step in this process is calculating the average of the 4 weeks of data:

6 $${E}_{mean}(t)=\frac{1}{4}\mathop{\sum }\limits_{i=1}^{4}{E}_{i}(t)$$

where *E*_*i*_(*t*) is the electricity consumption of each week and i is the number of weeks used in the calculation (*i* = 4). Four weeks are the approximate maximum number of weeks such that by averaging them, the significant spikes occurring during a day will not be missed. Substantially beyond 4 weeks, the extreme values will have been removed by the averaging and adding extra weeks will not make a difference.

For the next step, we apply the EMD on the averaged weekly electricity demand profile *E*_*mean*_. In the case of the NOVAREF data, 17 individual and independent modes are extracted. Finally to calculate the ALP, we sum the last N low-frequency modes.

7 $$ALP=\mathop{\sum }\limits_{i=1}^{N}{M}_{i+s}$$

where *M*_1+*s*_ is the highest-frequency mode still to be included. In the case of the NOVAREF data, we can have, as an example, *N* = 8 and *s* = 9, hence all modes from *i* + *s* = 10 up to *i* + *s* = 17 are summed up to obtain the ALP.

To determine the optimal number of low modes, i.e. *N*_*optimal*_, to sum and quantify the performance of the optimised ALP, we apply the mean-squared error (MSE) on the validation set. The MSE measures the average squared difference between the estimated values and the actual values^69^ and we calculate it as follows:

8 $$MSE=\frac{1}{L}\mathop{\sum }\limits_{i=1}^{L}{[P({{{{{{{\rm{ALP}}}}}}}},{t}_{i})-P({{{{{{{\rm{measurement}}}}}}}},{t}_{i})]}^{2}$$

where *P*(ALP, *t*_*i*_) is the ALP obtained from summing up the certain N modes in the training set, *P*(measurement, *t*_*i*_) is the measured consumption time series belonging to the validation set. *t*_*i*_ is the time in seconds, 0 < i < L and L is the length of data used to normalise the ALP. For the NOVAREF dataset L is 7 days of data in 2 s increments.

The MSE is calculated for both the ALP (*MSE*_*ALP*_) and the H0 SLP (*MSE*_*H*0*SLP*_) and compared (see Fig. 3 in “Demand trend: mode decomposition”). The comparison reveals that:

9 $$MS{E}_{ALP} \; < \; MS{E}_{H0SLP}$$

for 5 < *N* < 12. Regardless of the number of modes summed to create the ALP there is always a minimum which fulfils the *MSE*_*ALP*_ < *MSE*_*H*0*SLP*_ condition (see Fig. 3b). This minimum gives the optimal number of low modes, i.e., *N*_*optimal*_, that must be summed to create the optimal ALP for a given set of chronologically consecutive 4 weeks. This minimum varies with the set of weeks used, though for the NOVAREF dataset *N*_*optimal*_ = 7 for the majority of the weeks investigated. A more detailed analysis of the model training, validation and test datasets used, can be found in Supplementary Note 3.

The non-randomness of our results  was also verified by calculating the fraction of the *MES*_*ALP*_ and the *MSE*_*H*0*SLP*_

10 $$MS{E}_{fraction}=\frac{MS{E}_{ALP}}{MS{E}_{H0SLP}}$$

for the *N* ≈ *N*_*optimal*_, *MSE*_*fraction*_ < 1.

## Supplementary information

Supplementary Information

## Data Availability

The ADRES household consumption data were provided by the TU Wien and are available for research purposes upon request https://www.ea.tuwien.ac.at/projects/adres_concept/EN/. The IDEAL household dataset is available in https://datashare.ed.ac.uk/handle/10283/3647?show=full. The ENERA household consumption data that support the findings of this study were made available by the DLR Institute for Networked Energy Systems and the EWE, respectively. Restrictions apply to their availability as they were used under license for the current study, and so are not publicly available. The data are, however, available from the authors upon reasonable request and with permission of the DLR Institute for Networked Energy Systems and the EWE. The NOVAREF and IDEAL household consumption data that support the findings of this study are available at https://osf.io/yu2dm/?view_only=685370675ca145eb88234031158fc32c for download under the CC-BY-4.0 license.

## Code availability

The code used to calculate the EMD, the ALP and the stochastic fluctuation analysis and generation is available for download on the OSF: https://osf.io/yu2dm/?view_only=685370675ca145eb88234031158fc32c, under the CC-BY-4.0 license.
