# Supplementary material for: Data-driven load profiles and the dynamics of residential electricity consumption
Source: Nat Commun. 2022 Aug 6;13:4593. doi: 10.1038/s41467-022-31942-9 (PMC9357012; doi:10.1038/s41467-022-31942-9)
Supplement: Supplementary file 1 — Supplementary Information [file 41467_2022_31942_MOESM1_ESM.pdf]

Supplementary Information accompanying the manuscript
Data-driven load profiles and the dynamics of residential electricity consumption

Mehrnaz Anvari,[1,*] Elisavet Proedrou,[2,*] Benjamin Schäfer,[3,4,5,*]
Christian Beck,[3,6] Holger Kantz,[7] and Marc Timme[8]

[1] *Potsdam Institute for Climate Impact Research (PIK), Member of the Leibniz Association, P.O. Box 60 12 03, D-14412 Potsdam, Germany*
[2] *DLR Institute for Networked Energy Systems, Oldenburg, Germany*
[3] *School of Mathematical Sciences, Queen Mary University of London, United Kingdom*
[4] *Faculty of Science and Technology, Norwegian University of Life Sciences, 1432 s, Norway*
[5] *Institute for Automation and Applied Informatics, Karlsruhe Institute for Technology, Germany*
[6] *The Alan Turing Institute, London, United Kingdom*
[7] *Max Planck Institute for the Physics of Complex Systems, D-01187 Dresden, Germany*
[8] *Chair for Network Dynamics, Center for Advancing Electronics Dresden (cfaed) and Institute for Theoretical Physics, Technical University of Dresden, 01062 Dresden, Germany*

Within this Supplementary Information, we provide additional evidence and material supporting the narrative and conclusions of the main text. In particular, we provide details on the data used, explain the detrending in more detail and provide in-depth analysis complementing the fluctuation analysis from the main text.

* contributed equally

Supplementary Note 1

Existing load profile models

As mentioned in Section *Complex demand dynamics – the necessity of new load profiles* many different residential load profile models exist (for a detailed analysis see [1]). Demand side management models, whose aim is to predict the electricity demand of houses in advance in order to be able to supply them with the required electricity, are a subsection of residential load profile models. There are many different types of models which can predict the load profiles at seconds, hours, days and months in advance, with varied degrees of success. The ones that are of interest to this paper are those models that can generate residential electricity load profiles of at least 24 hours with a temporal resolution of the order of hours to seconds. At present only eighteen such models exist and none have a temporal resolution higher than 10 seconds. A table containing all such models can be seen in Supplementary Table IV.

Supplementary Table I. **Load profile 24 hour model categorisation table.** The models are sorted according to their sampling rate and year of publication. (Source: [1])

Authors	Year	Sampling Rate	Modelling Techniques
Bartels et al.	1992	1 hour	[2]
Yao et al.	2005	1 hour	[3]
Paatero et al.	2006	1 hour	[4]
Ren et al.	2012	1 hour	[5]
Shao et al.	2013	1 hour	[6]
Gottwalt et al.	2018	1 hour	[7]
Capasso et al.	1994	15 minutes	[8]
Alzate et al.	2014	15 minutes	[9]
Collin et al.	2014	10 minutes	[10]
Gao et al.	2016	8.5 minutes	[11]
Armstrong et al.	2009	5 minutes	[12]
Gruber et al.	2012	1 minute	[13]
Bajada et al.	2013	1 minute	[14]
Marszal-Pomianowska et al.	2016	1 minute	[15]
McKenna et al.	2016	1 minute	[16], [17]
Neue et al.	2016	1 minute	[18]
Dickert et al.	2010	30 s	[19]
Fischer et al.	2015	10 s	[20]

Publicly available measured residential consumption data set

There is only a small number of publicly available measured electricity consumption data sets at the moment and a complete list can be seen in Supplementary Table II. All but one of the data sets were measured in the last two decades (after 2000). They are all publicly available for research purposes and the data can be downloaded directly from the website or by contacting the author.

Data sets used in the paper

Due to the low number of publicly available data sets (see Supplementary Table II) and the fact that all of them lacked the high temporal resolution that we needed or lacked the length of measurement/high enough number of houses we required, we were forced to use three non-publicly available data sets and one publicly available data set: (a) the Austrian ADRES data set [44], (b) the German NOVAREF data set [52], (c) a small subsection of the German ENERA data set [53], and a part of UK IDEAL household energy data set [54] and compared them to the publicly available, standard load profile H0 SLP. A presentation of each data set can be seen below.

Supplementary Table II. **Publicly available data sets of residential electricity consumption.** This table presents all the publicly available residential electricity consumption data sets which contain measurements of a minimum of 24 hours. They are sorted by year of publication and include the sampling rate, the measurement period, the features of each data set and the country where the data were measured. The features available are A1 = aggregate consumption of single household/s, A2 = electric car, A3 = individual circuits consumption, A4 = occupancy status, A5 = PV generation, A6 = micro-wind generation, A7 = individual appliances consumption, A8 = indoor temperature, A9 = outdoor temperature, A10 = building, room and appliance characteristics.

#	Acronym	Year	Sampling Rate	Submeters	Features	# Houses	Period	Country
1	SERL [21]	2020	24 h & 30 min	-	A1	1770	2019-2020	UK
2	CRHLL [22]	2013	1 hour	10	A1, A3, A7	16	1 year	USA
3	HUE [23]	2019	1 hour	-	A1	28	1-2 years	Canada
4	Ausgrid Solar Home Electricity [24]	2010	30 min	-	A5	300	2011-2013	Australia
5	ISSDA Smart Meter data set [25]	2012	30 min	-	A1	4225	2009 - 2010	Ireland
6	LCLdToU [26]	2016	30 min	-	A1	5567	2013	UK
7	EDR Project [27]	2018	30 min	-	A1	16249	2007-2010	UK
9	H0 SLP [28], [29]	1999	15 min	Aggregated	A1	332	1970 - 1999	Germany
10	IZES [30]	2010	15 min	-	A1	497	2010	Germany
11	IEEE PES-ISS [31]	2015	15 - 5 min	-	A1, A2	10	8 - 30 days	USA, Brazil
12	Smart* (UMSM) [32]	2013	15 - 1 min	42	A1, A3, A4, A5, A6	400 & 7	2014-16	USA
13	IHEPCDS [33]	2012	1 min	3	A1, A3	1	4 years	France
14	SustDataED [34]	2012	1 min	24	A1, A7	50	2010-present	Portugal
15	MEULPv1 [35]	2012	1 min	8	A1, A3	12	> 1 year	Canada
16	iAWE [36]	2013	1 min	33	A1, A3, A7	1	73 days	India
17	AMPDs [37]	2013	1 min	21	A1	1	2 years	Canada
18	MEULPv2 [38]	2017	1 min	5 groups	A1, A2	12	1 year	Canada
19	REDD [39]	2011	10 & 3 s	24	A1, A7	6	several weeks	USA
21	REFIT [40]	2017	8 s	9	A1, A7	20	2 years	UK
22	Tracebase [41]	2012	2 s	158	A7	15	24 hours	Germany,Australia
23	ECO [42]	2014	1 s	6	A1, A3, A4	6	8 months	Switzerland
24	GREEND [43]	2014	1 s	9	A1, A7	8	1 year	Austria, Italy
25	ADRES [44]	2015	1 s	Aggregated	A1	39	14 days	Austria
26	DRED [45]	2015	1 s	13	A1, A8, A9	1	6 months	Netherlands
22	RAE [46]	2016	1 s	24	A1	2	9 - 63 days	USA
21	UK-DALE [47]	2017	6 s & 16 kHz	4	A1, A7	5	4,3 years	UK
22	BLUED [48]	2012	12 Hz	Aggregated	A2, A7	1	1 week	USA
23	ENERTALK [49]	2019	15 - 11 Hz	1-7	A1, A7	22	30 - 122 days	Korea
24	NOVAREF [50]	2020	2 s	-	A1	12	1 year	Germany
25	IDEAL [51]	2020	1 s	19	A1, A7, A10	39 - 255	20 months	UK

ADRES data set

The ADRES data set was created during the ADRES project, which was funded by the Austrian Climate and Energy Fund under the program ”ENERGIE DER ZUKUNFT” [44]. During the project, the residential electricity consumption of 39 households (21 single family houses, 8 apartment buildings and 10 apartments) located in Upper-Austria, were recorded between 2009 and 2010, as part of a cooperation between the Energie AG Oberöstereich and the TU Wien. The electricity consumption of each house was measured for the duration of two weeks in the winter (September – December 2009), and two weeks in the summer (May – October 2010).

The data set consists of high-resolution (sampling rate = 1 Hz)) active and reactive power data, as well as per

phase voltage measurements. At the end of the project, the anonymised electricity consumption of two weeks of measurements of 30 houses (one week in the winter and one week in the summer) were made available on request for use in research projects. The data set can be seen in Supplementary Fig. 1.

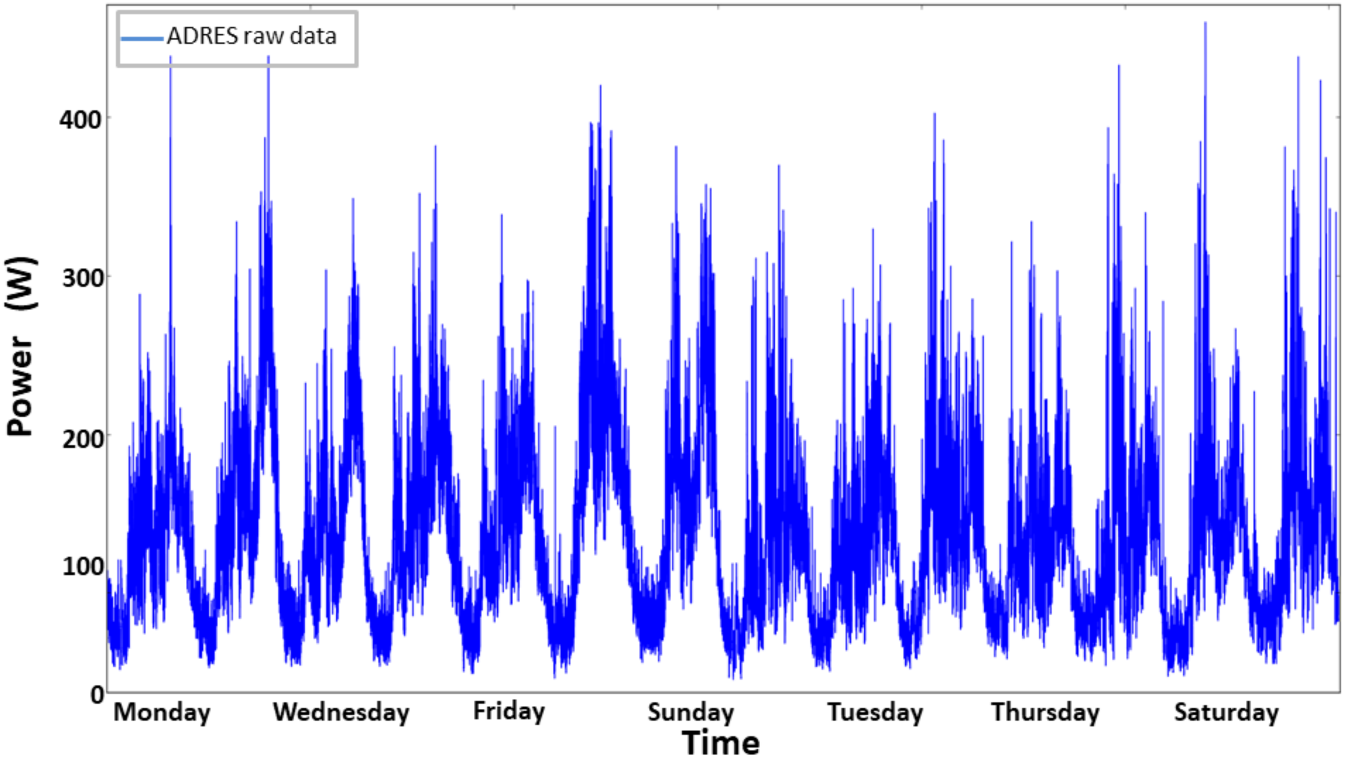

Supplementary Figure 1. **ADRES data set** The averaged load profile of the 30 ADRES houses for one week in the winter (first half of the plot) and one week in the Summer (second half of the plot.)

NOVAREF data set

The NOVAREF data set was created during the NOVAREF project, which was funded by the German Federal Ministry for Economic Affairs and Energy (BMWi). The residential electricity consumption of twelve houses (7 conventional houses and 5 low energy houses) in the northwest city of Germany, Oldenburg, were recorded between 2013 and 2016.

The data set consists of a year's worth of measurements of high-resolution (2 s) active power of the twelve houses and has a sampling rate of 0.5 Hz. At the end of the project, the anonymised electricity consumption of the twelve houses were made available for use to the BMWi funded DYNAMOS research project. Each house was measured for a single calendar year, between 00:00:00 01.01 and 23:59:59 31.12. The measurement were carried out between 2013 and 2016.

Out of the 52 weeks of data that were made available to us 16 were discarded due to the presence of datagaps caused by failures of the measuring equipment and power outages. Some of the data gaps lasted only a day, while occasionally an entire week might be missing. This left us with 36 weeks (10 months & 28 days) of usable data to work with. Partial results are presented here. In Supplementary Fig. 2, one year average of the NOVAREF load profile data is shown.

ENERA data set

The ENERA data set used here is a small subset of the data collected by the EWE AG during the ENERA project, which was funded by the BMWi SINTEG funding program. The project is ongoing at the time of publication of this paper. During the project the electricity consumption of more than 200 single family houses in the northwest

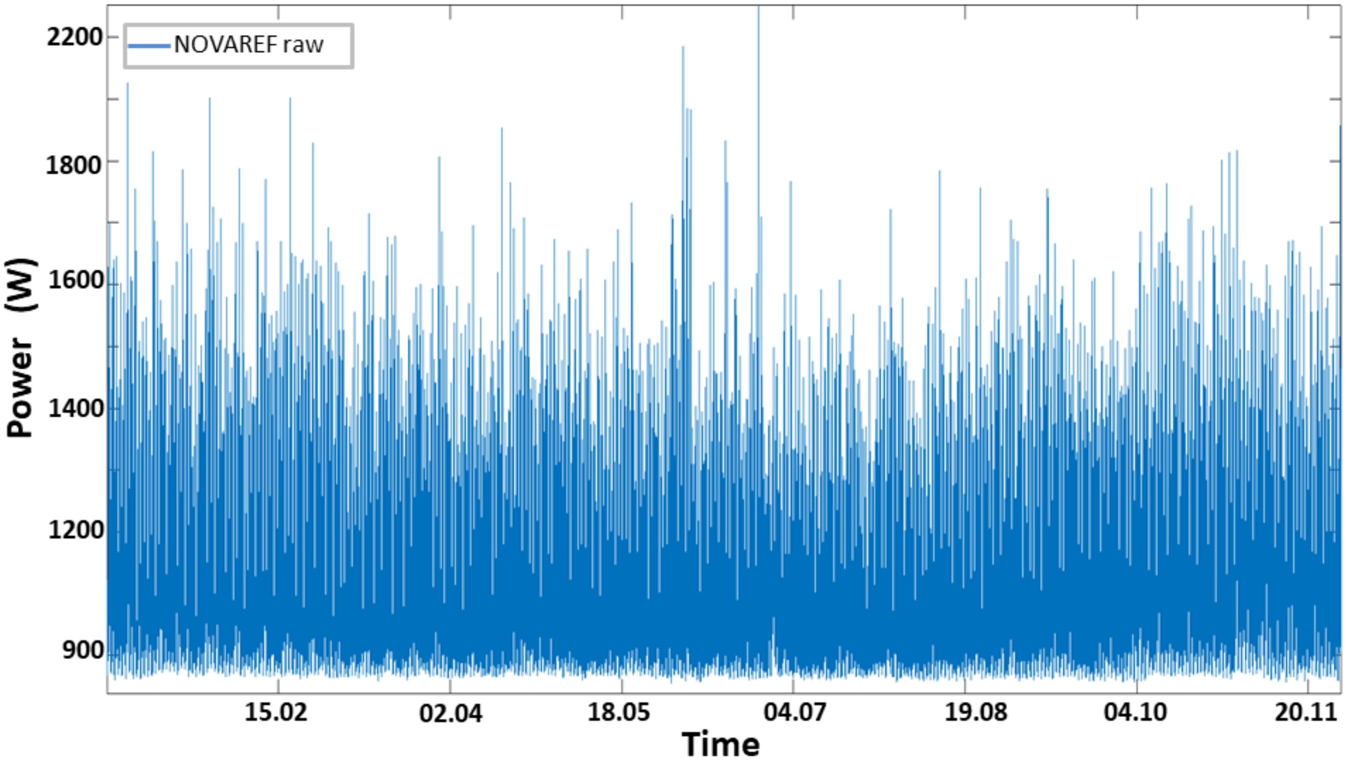

Supplementary Figure 2. **NOVAREF data set** The load time series averaged over the 12 NOVAREF houses for one year.

city of Germany, Oldenburg were recorded. We were given access to the electricity consumption of 70 houses. Each residential electricity consumption had a length of 6 weeks, recorded from 01.08.2019 to 13.09.2019, and the temporal resolution is 1 second. Due to gaps in the measurements 72 of the load profiles had to be rejected. In Supplementary Fig. 3, the averaged consumption data of those 70 houses for the entire 6 weeks is shown. As can be seen significant spikes are still present in the averaged load profile despite the averaging. In Supplementary Fig. 4, the averaged consumption data of a single day (02.08.2019) of those 70 houses is shown. From the figure it is clear that significant spikes are still present in the averaged daily load profile, when we compared it with the H0 SLP in the main text.

IDEAL data set

The IDEAL data set used here was collected as part of two EPSRC-funded projects, IDEAL and BIGSMALL [54]. The electricity consumption of 255 households from Edinburgh and the nearby regions of the Lothians and south Fife, in Scotland, UK, were measured from 10.8.2016 to 30.06.2018, with a temporal resolution of 1 second. To make our results comparable to those of the NOVAREF data we chose 11 households whose electricity consumption was measured simultaneously for approximately 4 months between November 2017 and March 2018. The data set is available at [55]. In Supplementary Fig.5 the averaged consumption data of 11 homes for 20 days is shown.

BDEW Standard Load Profile

The H0 SLP was created by the German Federal Association of Energy and Water Management (Bundesverband der Energie- und Wasserwirtschaft (BDEW) e.V.), in cooperation with the chair of the Energy Economy of the Brandenburg University of Technology Cottbus, to help the electricity providers to forecast their costumer's yearly electricity consumption on the basis of empirical values and knowledge of the customers/customer groups, and adjust their network management to the expected load flow. The majority of the data used to create the data set were measured before 1970. To create the standard load profile they collected all the residential electricity consumption data sets measured in the decades before 1999 [29]. After filtering the data sets for measurement gaps, measurement errors and the length of measurement (each data set had to have been measured for at least one calendar year), they

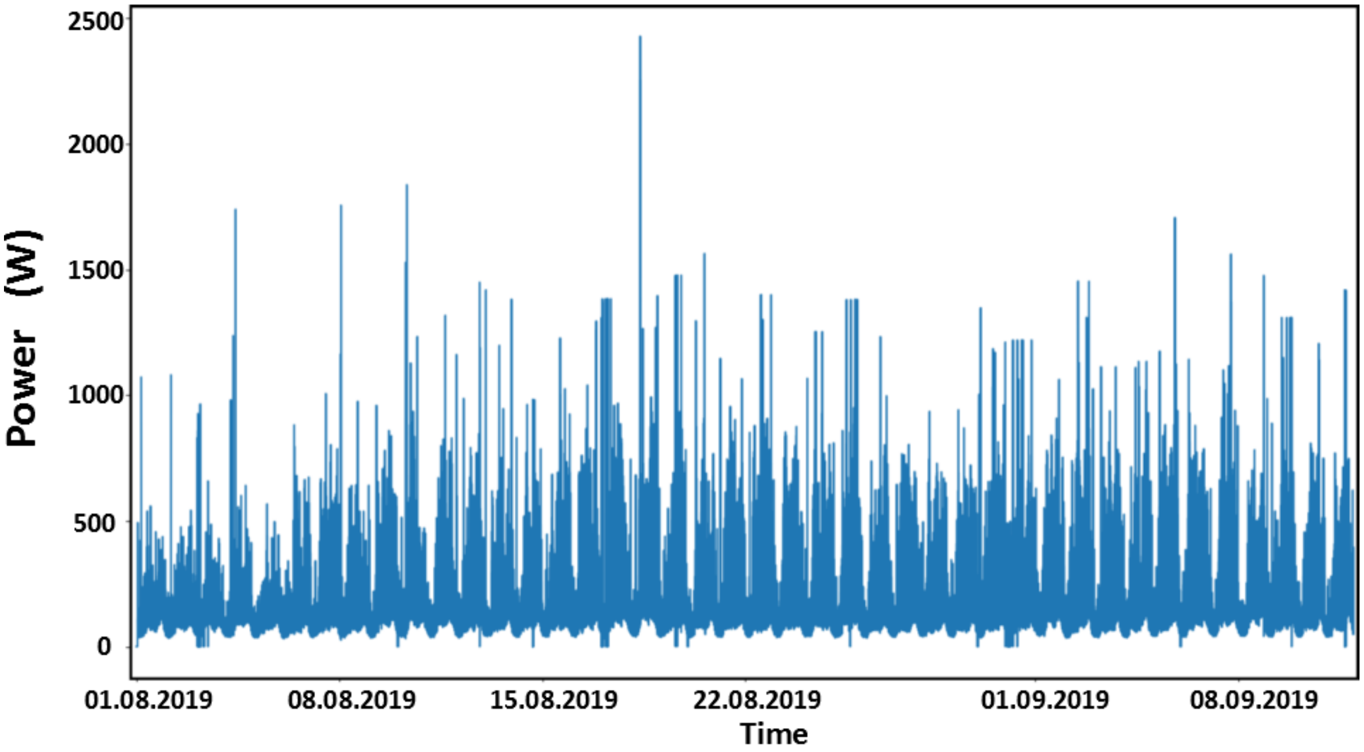

Supplementary Figure 3. **ENERA data set** The load time series averaged over the 70 ENERA houses for 6 weeks measured between 01.08.2019 - 11.09.2019.

isolated the measurements from 332 houses. The older data sets had a sampling rate of 1 hour, while the newer data sets, measured in 1981, 1982, 1986, 1987, 1995, 1996 and 1997 had a sampling rate of 15 minutes. The hourly data sets were then upsampled to 15 minutes and characteristic winter, spring/autumn and summer weekdays and weekend days were determined [29]. These characteristic days were then normalised to a yearly consumption of 1000 kWh/year, so that they would provide the average expected quarter-hourly electricity consumption when a consumer consumes 1000 kWh in a single calendar year [56], see Supplementary Fig. 6. It is worth noting that the Austrian equivalent of the BDEW (the Energie-Control Austria für die Regulierung der Elektrizitäts- und Erdgaswirtschaft) uses the BDEW SLP as its standard load profile [57]. Because the BDEW SLP is known as H0 SLP in the engineering community, we will use the term H0 SLP to refer to it in the rest of the paper. The yearly residential standard load profile (H0 SLP) is visible in Supplementary Fig. 7, where the yearly variation is clearly visible, with high electricity consumption in the winter and low electricity consumption in the summer. When it is compared to the electricity consumption of the ADRES and NOVAREF data sets, it is clear that they do not follow the same yearly consumption patterns and their winter electricity consumption does not appear to vary strongly with the time of the year.

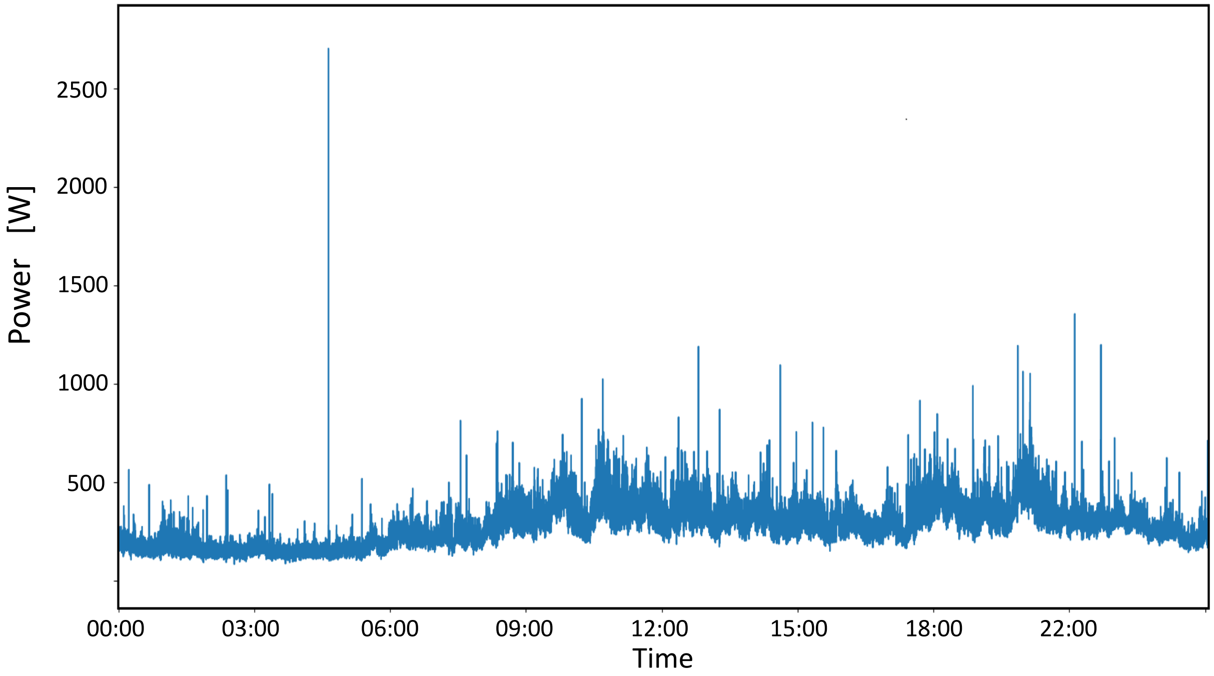

Supplementary Figure 4. **Daily load profile of 70 houses** Here the load profile of 70 houses measured during the ENERA project for one day (02.08.2019) is shown. Despite the averaging of the electricity consumption of 70 houses the significant spikes, due to the high temporal resolution of the data set, are still present in the load profile, when we compared it with the H0 SLP in the main text, (see Fig. 1).

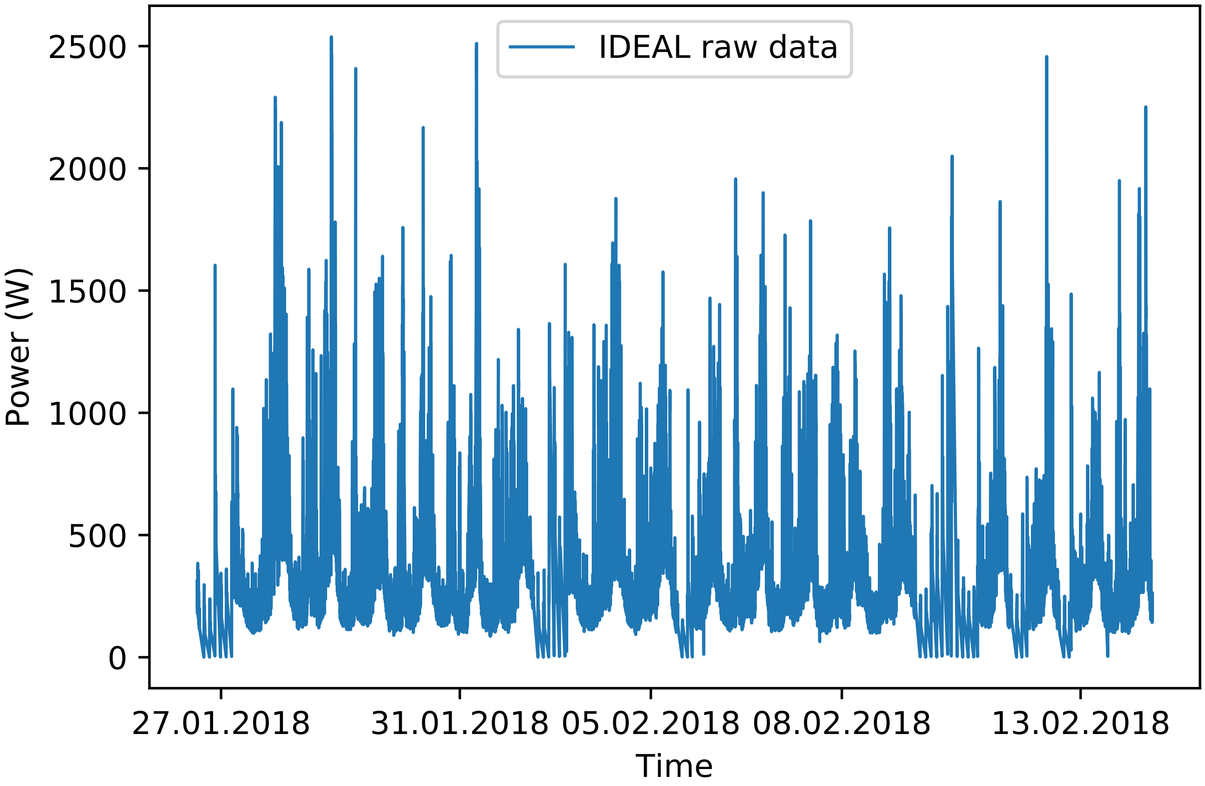

Supplementary Figure 5. **IDEAL data set** The load time series averaged over the 11 IDEAL houses for 20 days measured between 27.01.2018 - 15.02.2018.

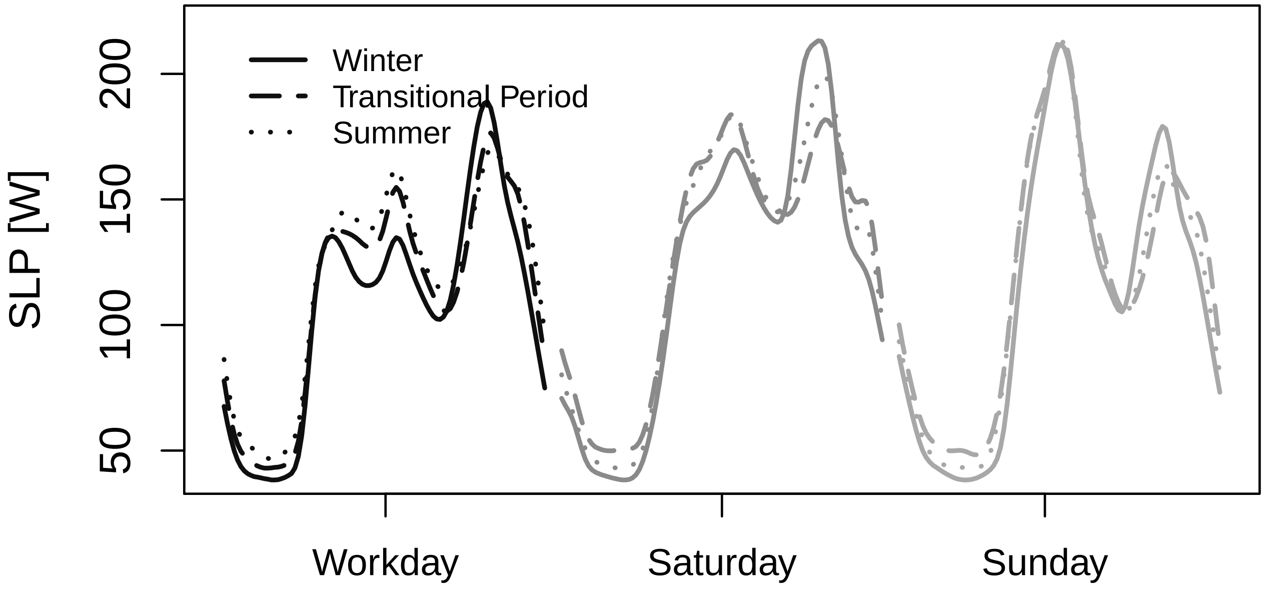

Supplementary Figure 6. **BDEW Standard Load Profile** From left to right H0 SLP belonging to workdays, Saturdays and Sundays has been respectively shown. The changes of H0 SLP during winter and summer, as well as transitional period, i.e. spring and autumn, have been respectively demonstrated by solid, dashed and dotted lines.

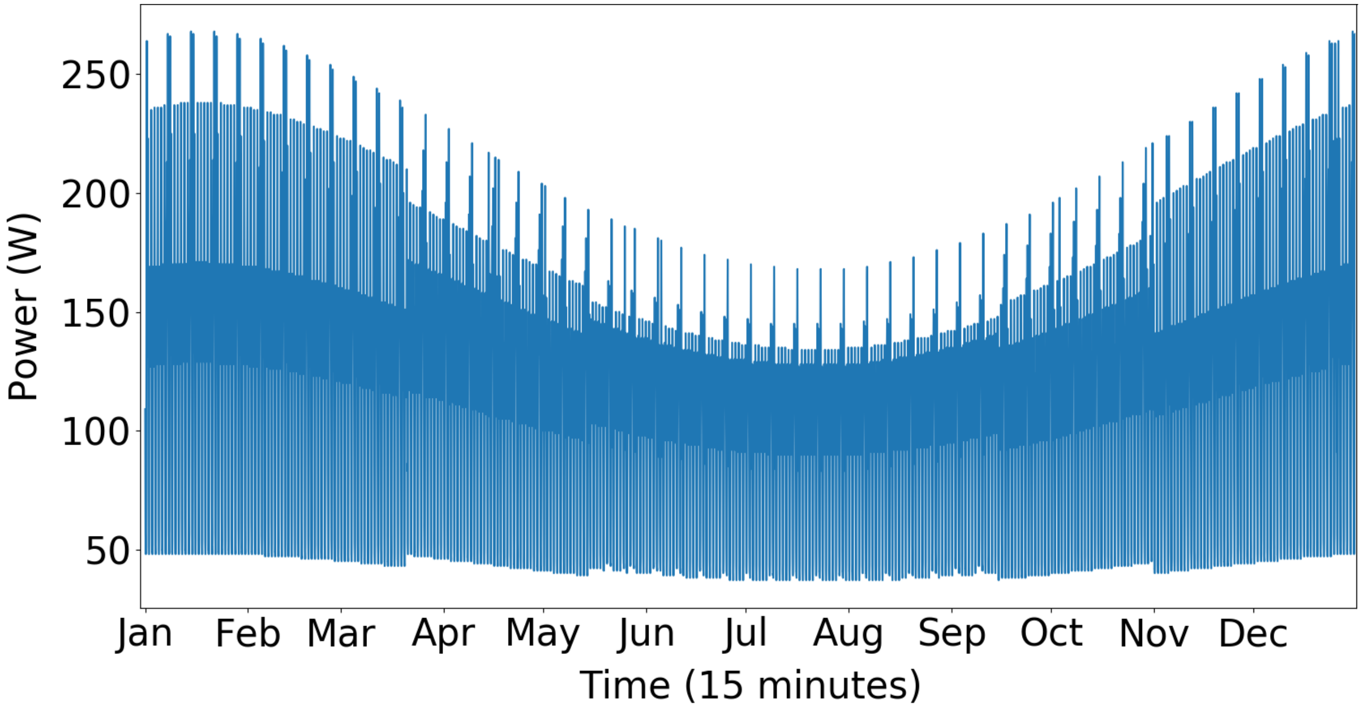

Supplementary Figure 7. **BDEW Standard Load Profile** The yearly load profile H0 SLP created by the BDEW. The darker areas in the plot are a artefact of the plotting and have no meaning. The profile is characterised by high electricity consumption in the winter and low electricity consumption in the summer. The spikes indicate weekends, when the electricity consumption is higher and is also characterised by a different profile on both Saturdays and Sundays as is visible in Supplementary Fig. 6. The gaps between the spikes are caused by the same week day profile repeated for each of the 5 days of the week (Monday-Friday). The gap between the spikes appears because the electricity consumption during a weekday is lower than the weekend electricity consumption.

Supplementary Note 2

Additional load measurements

In the main text in Section *Complex demand dynamics – the necessity of new load profiles*, we discussed the influence of new power generation, such as PV, on the load profile. Here, in Supplementary Fig. 8, we present how the simultaneous charging of 12 identical e-cars (which we assume are owned by each of the 12 NOVAREF households) will affect the averaged household profile. All cars have Level 1 chargers (of the SAE J1772 standard) which operate at 1.92 kW [58]. The electric car charging data were obtained from [58], which provides simulated consumption data with 10 minutes temporal resolution, based on the type of the battery used in each electric car. It is clear from Supplementary Fig. 8, that during the charging phase of the plug-in car there is a large increase in the power consumption, which we have superimposed to the previously existing fluctuations. Therefore, to predict the load profile precisely, one has to know the time duration of the plug-in charging, the model of the battery and the number of electric cars in each house. If multiple households in the same distribution or micro grid charge their cars at the same time, this would lead to coincident consumption and contribute significantly towards large consumption peaks. It should be noted that the increase of the required demand during the charging of e-cars is reported to be the cause of the local transformers ageing [59]. We explain the coincident consumption in the following paragraph.

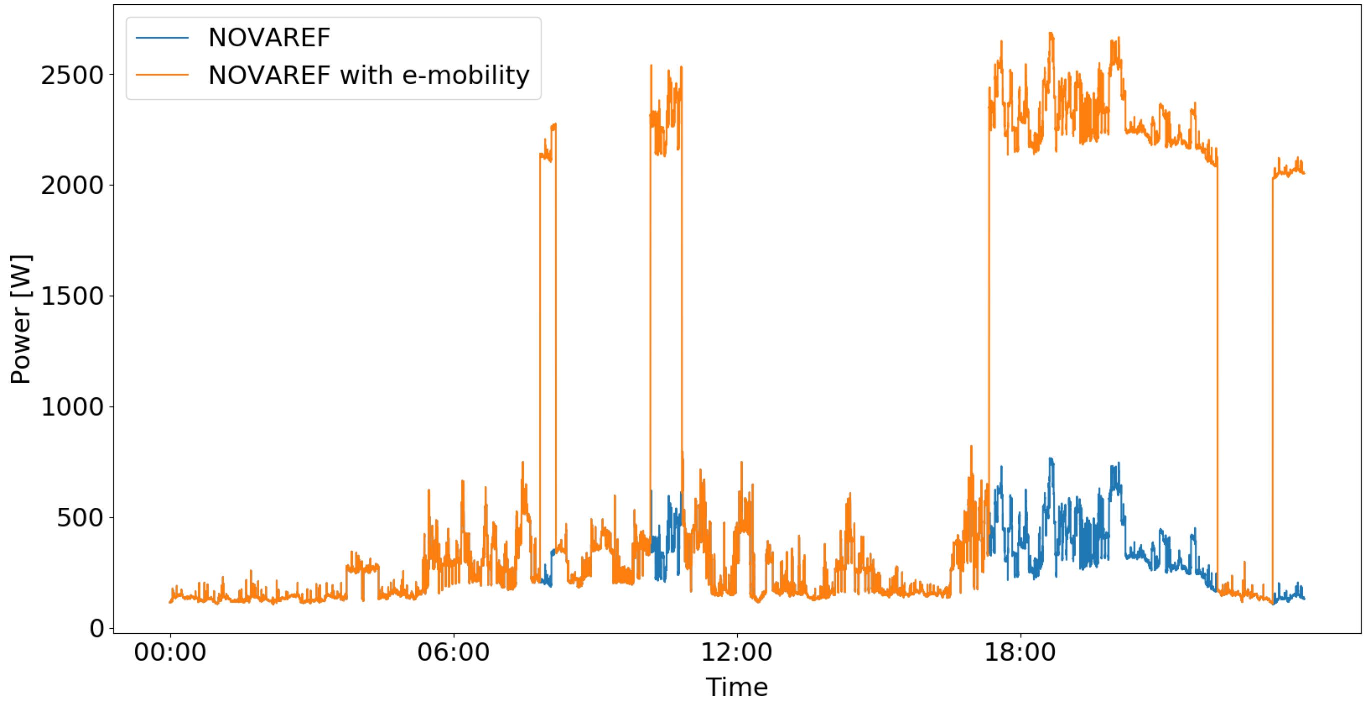

Supplementary Figure 8. **E-mobility effects on the averaged load profile of the 12 NOVAREF houses** We demonstrate how the simultaneous charging of 12 identical cars (which we assume belong to the 12 families/individuals living in the 12 NOVAREF houses) affects the electricity consumption. As seen, when the cars are plugged-in a sharp jump in the electricity consumption is visible. The blue line indicates the averaged load profile of the 12 houses. The orange line indicates how the NOVAREF averaged electricity consumption would be altered by the simultaneously charging e-cars. This is not such an absurd assumption. The traffic jams during the morning and evening rush hours in Germany indicate that a great many people leave their work at more or less the same time. If they also happen to live close by, they will in all probability plug in their car to charge the moment they arrive to their house.

Looking at the daily load profile of an individual household, it is clear that the power consumption is very variable (see Fig. 1(c) in the main text) and mostly depends on the life style of the household residents [60]. Moreover, if we consider the average power consumption of several households and look at the averaged load profile instead of the load profile of individual households, we can see that, apart from the fluctuations, the trend of the averaged load profile, which to some extend follows the H0 SLP, is not recognisable in the single household load profile, see Supplementary Fig. 8. When we began this research we expected that the stochastic fluctuations present in the averaged load profile of the NOVAREF, ADRES and ENERA households could be described using white noise. However, in Section *Demand fluctuations: Stochastic model* we showed that these fluctuations are intermittent and cannot be described by Gaussian

noise. One reason for the non-Gaussianity could be the interaction between households, which can be explained by the diversity factor (see Supplementary Fig. 9). To calculate the diversity factor, we first find the maximum of the energy consumption in 15 minutes intervals during a day for each house and then we sum these maxima up. The resulting values are known as maximum coincident demand, P_{cd}. In the next step we find the maximum consumed energy of each house during a day and then sum these maxima up. This calculated value is known as maximum non-coincident demand, P_{ncd} [15]. Finally, to find the diversity factor, we divide the P_{cd} by P_{ncd}. The diversity factor has values between 0 and 1. The larger the diversity factor, the more the households interact. In Supplementary Fig. 9, we show the diversity factor for the ADRES, NOVAREF as well as ENERA data which are composed of 30, 12 and 70 houses, respectively. Looking at Supplementary Fig. 9 we can see that in the fourteen selected days, there exist several time intervals during a single day where the value of the diversity factor exceeds 0.3, which proves **to** us that there are interactions between the households in all three data sets. However, the value of the diversity factor can decrease as the number of houses increases. If we look at the diversity factor of the ENERA data set we can see that the number of days where the diversity factor is higher than 0.4 has decreased compared to the ADRES and NOVAREF data sets, but have not disappeared altogether. Indeed some days and some time intervals that the interaction is significant still exist. So in the cases of decentralised networks or localised microgrids, where the number of houses is expected to be lower, we can expect that it will not be possible to smooth the high frequency fluctuations by simply averaging the electricity consumption of the houses.

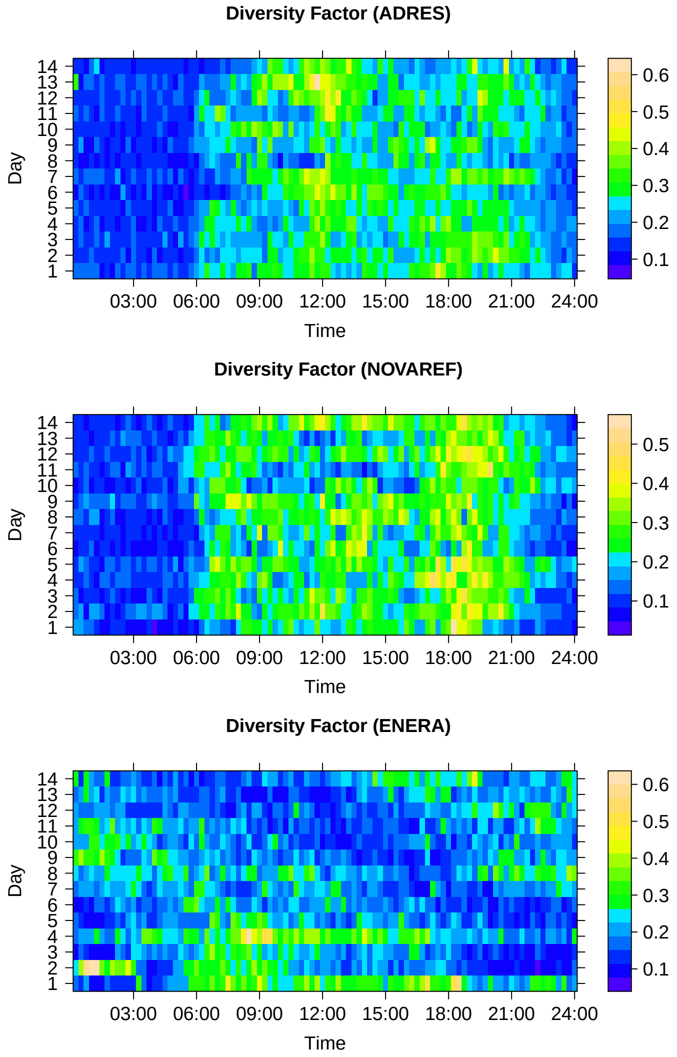

Supplementary Figure 9. **Diversity factor** The diversity factor for the ADRES, NOVAREF and ENERA data sets which are composed of 30, 12 and 70 houses, respectively. It should be noticed that the diversity factor of ADRES data set, which has been shown here, and the one in Fig. 2(a) in the main text is completely same and, just, the colour spectrum of the colour bars is different.

Supplementary Note 3

Trend extraction

Here we provide a more detailed presentation of the adaptive time-frequency data analysis method used to generate the ALP for the NOVAREF and IDEAL data sets. The process can be used to estimate the ALP for any electricity consumption data set with a high temporal resolution of at least 1 minute and, preferably, without data gaps. If a considered data set includes a lot of data gaps the EMD method may not be able to extract modes accurately.

The first step in the analysis of the NOVAREF data is to average the recorded electricity consumption over all houses and remove all weeks with data gaps. This creates a single 34 weeks long electricity consumption data set. We then divide this data set into Monday to Monday weeks.

As a further step we split 34 weeks into two seasonal data sets: (i) a winter data set from 01.07 to 06.24; (ii) a summer data set from 07.01 to 12.23. It should be noted that the electricity consumption of the entire set of houses has been measured during multiple years, where every single house has been recorded only for one year long period, e.g. the electricity consumption of house numbers one and two has been recorded in 2013, while house number 11 in 2016. Therefore, we average one year long electricity consumption over houses, assuming the monthly distribution of every house remains similar during the three years period.

Each of these seasonal data sets are then split into a: (i) training set; (ii) validation set and; (iii) test set. Our analysis has showed that the training set used to train the ALP should include 4 consecutive weeks. Here, it includes 01.07-14, 01.14-21, 02.04-11, 02.11-18 for the NOVAREF winter set, and 07.01-08, 07.08-15, 07.15-22, 07.29-08.05 for the NOVAREF summer set.

However, both the validation and test set can include randomly chosen weeks from the periods 03.11-06.24 and 08.05-12.23, respectively. The number of weeks that each set is composed of is listed in Supplementary Table III.

The subset of the IDEAL data set that we initially used for our analysis containing data gaps larger than three hours and hence required a special pre-processing: Weeks with data gaps smaller than three hours were isolated and, then, their gaps were filled through resampling and interpolation. This procedure, however resulted in large spikes appearing in the weekly EMD modes, which completely overshadowed other features of the data set and made it impossible to identify a reasonable ALP. As a result, it is impossible to isolate enough individual weeks to perform the same (weekly) analysis for IDEAL data, as we did with the NOVAREF one.

When analysing the IDEAL data set, instead of extracting the ALP for a weekly electricity consumption, we extract the ALP for a daily one. This has the advantage to demonstrate the applicability of the ALP approach not only as a weekly ALP but also as a daily one. Finally, after averaging the recorded electricity consumption without data gaps over all houses, we have a single 37 workdays long of electricity consumption in the time period of 11.13-11.17.2017 and 01.29-03.26.2018. We then split these days into training, validation and test sets. The training set should include seven days of either weekday or weekend day type. Here, the training set includes weekdays type that are chronologically ordered as follows: 01.29.2018, 01.30.2018, 01.31.2018, 02.01.2018, 02.05.2018, 02.16.2018, 02.19.2018. Similar to the NOVAREF analysis, validation and test can include randomly chosen days. The number of days each set is composed of is listed in Supplementary Table III.

Based on our experience in analysing NOVAREF data set, to extract a weekly ALP, the considered data set should be at least six weeks long, i.e. four weeks are required for training, one week for the validation and one week for the test (Please also see Supplementary Note 5 and Table IV). To extract a daily ALP for IDEAL data set, one needs at least nine days of either weekday or weekend type, i.e. seven days are required for training, one day for the validation and one day for the test. The minimum number of required weeks and days for the further analysis has been found empirically and the number might change for different data sets, in particular for regions with very pronounced seasonality [61].

Supplementary Table III. **Data distribution per set** The composition of the training, validation and test sets for each data set, together with their share of the total data (in parenthesis).

data set	Training Set	Validation Set	Test Set
NOVAREF winter	4 weeks (23.5 %)	9 weeks (53%)	4 weeks (23.5 %)
NOVAREF summer	4 weeks (23.5 %)	9 weeks (53%)	4 weeks (23.5 %)
IDEAL	7 days (18.9 %)	15 days (40.5%)	15 days (40.5%)

For the analysis of the NOVAREF data set we applied the EMD method on the entire 34 weeks data set in order to extract the multiple signals (modes) embedded in the averaged electricity consumption of the houses. Then, as discussed above, we separated the summer data from the winter one. In the next step we define the training, validation and test sets for each season. Differently, for the IDEAL data set we applied the EMD separately on each day after

the assignment of days to training, validation and test sets. It should be noted that the method used for the IDEAL data provides also accurate results. A short discussion on the subject is found in Supplemental Note 5.

We then follow the process described in Methods of the main text to determine the optimal number of modes ($N_{optimal}$) and, hence, the optimal ALP. Because this process is data-driven, the $N_{optimal}$ will vary depending on the training data set.

In the case of the NOVAREF data set, in both validation and test sets $N_{optimal} = 7$ is valid, whereas in the case of the IDEAL data set $N_{optimal}$ can change slightly depending on the day evaluated. Indeed, the $N_{optimal}$ can likely be different for distinct geographical regions and might also depend on the number of houses included in the demand profile. Due to the low number of highly resolved data sets available to us and their relatively short length, we did not investigate whether the number of days or weeks used to train the ALP affects the $N_{optimal}$.

Supplementary Note 4

Stochastic model and superstatistics

Here, we illustrate how the stochastic model describing the demand fluctuations is derived and how synthetic trajectories are generated. We also demonstrate that including more or fewer fluctuating modes into the trend or the fluctuations does not change the results substantially.

Similar to the EMD analysis, we mainly stick to the NOVAREF data set recorded in Oldenburg, Germany. While the data set covers a full year, we will analyse the first week within the data set in more detail. Results obtained are general and we later compare the synthetic model based on the first week with a completely different week.

Demand fluctuations

The demand data fluctuate rapidly on various time scales, as we see from inspecting the trajectory for the one week we study here, see Supplementary Fig. 10.

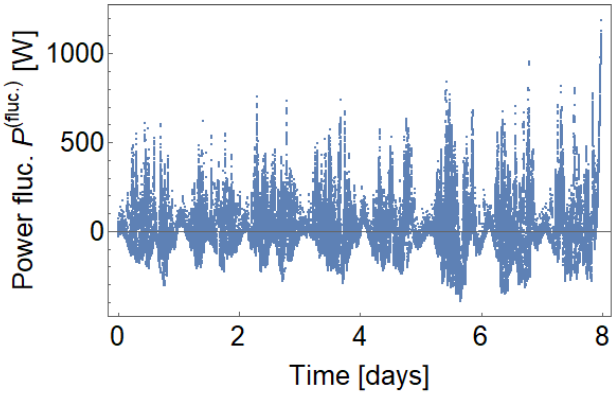

Supplementary Figure 10. **The consumption variation follows regular patterns on multiple time scales** We display the detrended data of the NOVAREF data set, where the detrending was carried out using the EMD method described in the main text.

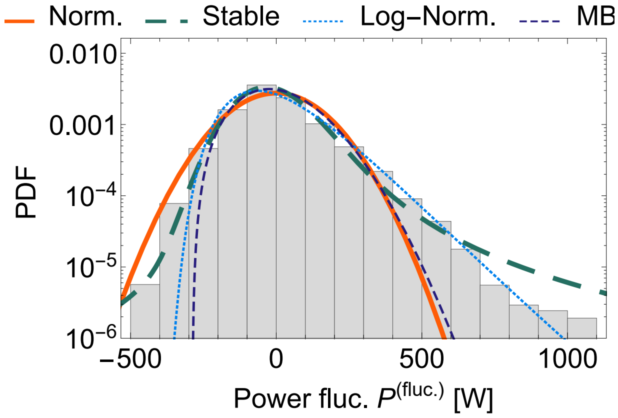

Supplementary Figure 11. **Basic distributions do not fit the observed histogram of the detrended data well** The normal distributions miss the asymmetry, stable distributions overestimate the tails substantially. Log-Normal gets the tails but is a bad description for negative values. Finally, Maxwell-Boltzmann distributions underestimate the tails but are a good initial guess as a local distribution for superstatistical applications. The histogram uses NOVAREF data from April 2018.

We continue the analysis of the fluctuation data by plotting the distribution of the demand variations in Supplementary Fig. 11. The data (histogram) is both heavy-tailed and significantly skewed, i.e. deviations to high values are more likely than deviations to low values. We fit the data using some basic distributions. A normal distribution is

a poor fit as it can neither describe the asymmetry nor any heavy tails. A stable distribution, hosting 4 parameters, is better in capturing the skewness. However, it overestimates the tails drastically. A Log-Normal distribution (shifted to the most negative value) captures the tail behavior but fails to describe the negative values. Finally, we consider a shifted Maxwell-Boltzmann distribution, whose PDF is given as

$$p(x) = \sqrt{\frac{2}{\pi}} \frac{(x-\mu_{MB})^2 \, \mathrm{e}^{-(x-\mu_{MB})^2/\left(2\sigma_{MB}^2\right)}}{\sigma_{MB}^3}, \tag{1}$$

for $x > \mu_{MB}$ and $p = 0$ otherwise. Here, $\mu_{MB} < 0$ denotes the shift of the zero-point necessary to account for negative values and σ_{MB} gives the scale parameter of the Maxwell-Boltzmann (MB) distribution. The MB distribution captures the general shape of the histogram in being asymmetric but does not quantitatively describe the tails very well.

We conclude that none of these distributions fit the data sufficiently well. Instead, we make use of the time-variations observed when inspecting the trajectories and apply a superstatistical description, using shifted Maxwell-Boltzmann distributions as the local distribution since these only use two parameters: the shape parameter σ_{MB} and the shift parameter μ_{MB}.

Superstatistics

Superstatistics uses the observed separation of time scales to split the data into simple local distributions, which aggregated, give a more complex global distribution, as we observe it in the real data. To apply superstatistics, we first have to determine the long time scale T on which the local distribution is properly described by a basic distribution. We test whether the data is described by local Gaussian or local Maxwell-Boltzmann distributions by calculating the "local" kurtosis κ and skewness β as

$$\kappa(\Delta t) = \frac{1}{t_{\max} - \Delta t} \int_0^{t_{\max}-\Delta t} \frac{\left\langle (x-\bar{x})^4 \right\rangle_{t_0,\Delta t}}{\left\langle (x-\bar{x})^2 \right\rangle_{t_0,\Delta t}^2} \mathrm{d}t_0, \tag{2}$$

$$\beta(\Delta t) = \frac{1}{t_{\max} - \Delta t} \int_0^{t_{\max}-\Delta t} \frac{\left\langle (x-\bar{x})^3 \right\rangle_{t_0,\Delta t}}{\left\langle (x-\bar{x})^2 \right\rangle_{t_0,\Delta t}^{3/2}} \mathrm{d}t_0, \tag{3}$$

where we vary the time window Δt, use the trajectory x, its mean $\bar{x}$ and the total length of the trajectory $t_{\max}$.

A simple distinction between Gaussian and Maxwell-Boltzmann distributions is that Gaussian distributions are symmetrical, i.e. have zero skewness, while Maxwell-Boltzmann distributions are skewed with skewness $\beta_{MB} \approx 0.486$. When computing the local skewness of the data, we find that indeed even for very small time windows Δt, the data is still skewed and is approximated by the Maxwell-Boltzmann skewness on the time scale of $\Delta t \sim 50 - 100s$, see Fig. 12. We will however not use the skewness but the kurtosis to determine the long time scale, as has been done in the literature.

Similar to the local skewness, we continue to compute the local kurtosis, see Fig. 13. We determine the large time scale T as the time window Δt so that $\kappa(\Delta t) = \kappa_{MB} \approx 3.108$, which is the value of the kurtosis for Maxwell-Boltzmann distributions. For the specific week chosen here, we find that $T \approx 2000s$. As a side remark, the long time scale T, determined using this method, does also depend on the particular data set chosen, i.e. a different week or a different region will return a different T. Still, to reproduce the approximate statistics observed in the data, the precise choice of T is not overly critical, see below. Future research could explicitly focus on the various time scales encoded in the different T, based on skewness and kurtosis analysis.

Finally, before we can use the stochastic model, we need additional information on the local Maxwell-Boltzmann distributions. In particular, we compute the autocorrelation $c(\delta t)$ as a function of the time lag δt. For many stochastic processes this autocorrelation decays exponentially $c(\delta t) \sim \exp(-\gamma_{MB}\delta t)$, as we also observe here in Supplementary Fig. 14. However, the decay constant γ_{MB} is not identical for all time windows but has its own distribution. We observe similar distributions when estimating the shift μ_{MB} and scale σ_{MB} of the local Maxwell-Boltzmann distributions, see Supplementary Fig. 15.

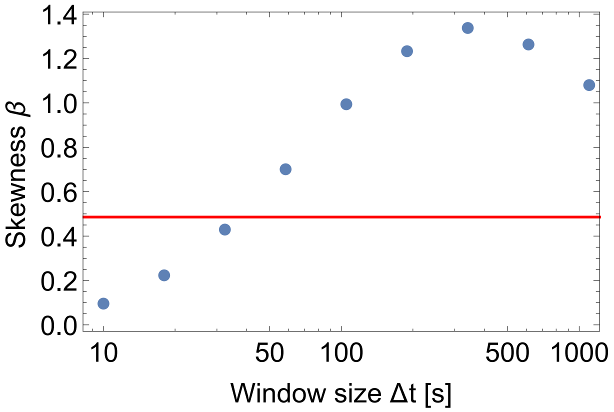

Supplementary Figure 12. **The skewness of the data is never zero, not even for very small time windows Δt** The red line gives the skewness of a Maxwell-Boltzmann distribution, while a Normal distribution would have zero skewness.

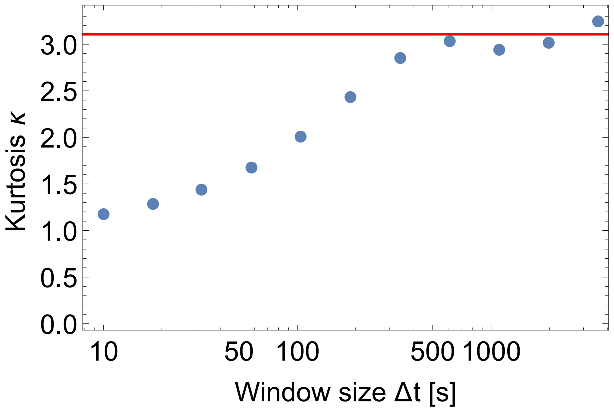

Supplementary Figure 13. **The long time scale T for the kurtosis is approximately 2000 seconds (1000 data points due to 2 second resolution)** We plot the local kurtosis as a function of the time window length Δt. The long time scale T is determined when the local kurtosis approximates the kurtosis of a Maxwell-Boltzmann distribution $\kappa_{MB} \approx 3.108$.

Formulating the stochastic model

Here, we formulate the stochastic model for the demand data, corresponding to the choice $J = 3$. For the more general models see the next section. We assume the power demand trajectory P is described by

$$P = P^{(\text{trend})}(t) + P^{(\text{fluc.})}(t), \tag{4}$$

where $P^{(\text{trend})}(t)$ gives the trend of the data as determined by a H0 SLP model or the EMD approach. Meanwhile, we have observed that the fluctuations of the demand $P^{(\text{fluc.})}(t)$ follow locally MB distributions. To obtain such local MB distributions, we apply the following set of stochastic equations,

$$P^{(\text{fluc.})}(t) = \sqrt{(x_1(t))^2 + (x_2(t))^2 + (x_3(t))^2} + \mu_{MB}, \tag{5}$$

$$\mathrm{d}x_i(t) = -\gamma x_i(t)\,\mathrm{d}t + \epsilon \mathrm{d}W_i, \tag{6}$$

where the x_i , with $i \in \{1, 2, 3\}$, follow a simple Ornstein-Uhlenbeck process, based on Wiener processes W_i. Hence, the x_i are identically but independently distributed Gaussian random variables with zero mean and exponentially decaying autocorrelation function with $c(\delta t) = \exp(-\gamma \delta t)$. Summing the squares of the Gaussian processes and then taking the square root leads to a Maxwell-Boltzmann distribution. To accommodate the observed shift in the empirical data, we have to add μ_{MB}.

Using insights from statistical mechanics and stochastic differential equations, we can easily find relations between the parameters ϵ, μ and γ on the one hand and σ_{MB}, μ_{MB} and γ_{MB} on the other: The shape parameter σ_{MB} of the Maxwell-Boltzmann distribution is identical with the standard deviation of the Gaussian processes: $\sigma = \frac{\epsilon}{\sqrt{2\gamma}} = \sigma_{MB}$,

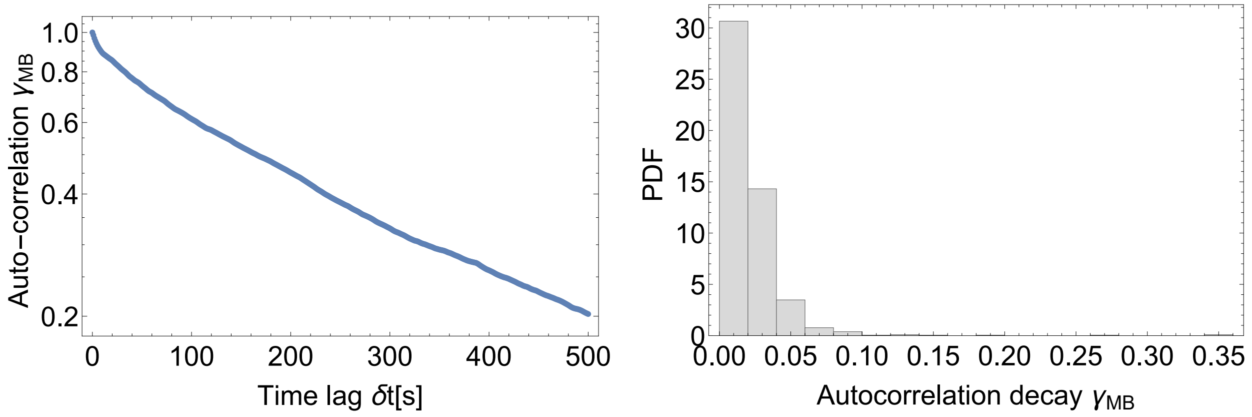

Supplementary Figure 14. **The autocorrelation decays exponentially** Left: We display the autocorrelation of the aggregated data for the NOVAREF data. The y-axis uses a log-scale so that the straight line indicates an exponential decay. Right: Fitting the exponential decay as $c = \exp(-\gamma_{MB}\delta t)$ gives a range of values of the exponential decay constant γ_{MB}. Taking the mean results in good agreement with the observed data.

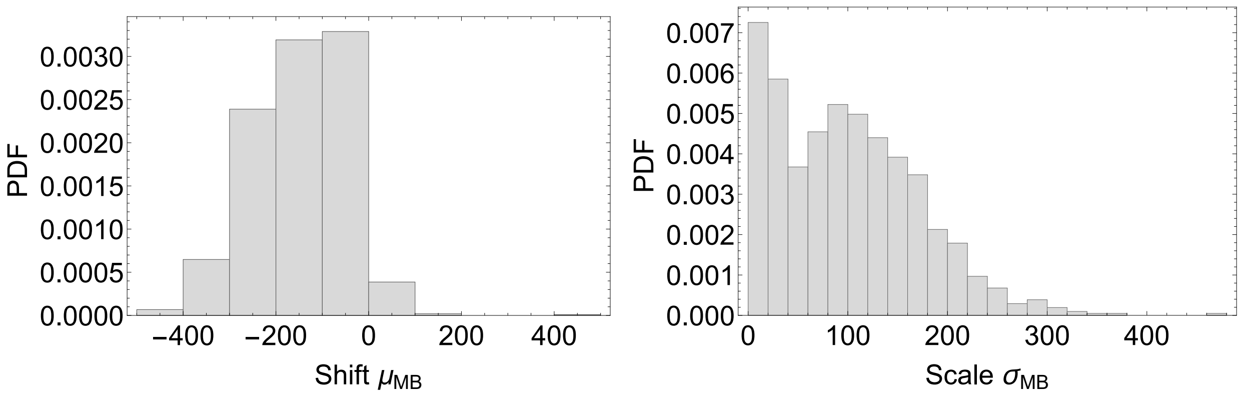

Supplementary Figure 15. **The local Maxwell-Boltzmann distributions have different values for their shifts μ_{MB} (left) and scale parameters σ_{MB} (right)** These parameters do not obviously follow a single simple distribution but might be approximated, e.g. by uniform, Gaussian or χ^2-distributions.

i.e. we obtain $\epsilon = \sqrt{2\gamma_{MB}}\sigma_{MB}$. The steady shift μ is identical with the shift from the Maxwell-Boltzmann distributions $\mu = \mu_{MB}$ and the same holds for the autocorrelation decay $\gamma = \gamma_{MB}$, resulting in the full model as

$$P^{(\text{fluc.})}(t) = \sqrt{(x_1(t))^2 + (x_2(t))^2 + (x_3(t))^2} + \mu_{MB}, \tag{7}$$

$$\mathrm{d}x_i(t) = -\gamma_{MB} x_i(t)\,\mathrm{d}t + \sqrt{2\gamma_{MB}}\sigma_{MB}\mathrm{d}W_i, \tag{8}$$

which describes each local Maxwell-Boltzmann distribution of a time window T.

The aggregated statistics is then generated by varying σ_{MB}, μ_{MB} and γ_{MB} over time: We pick the first interval $t_1 \in (0, T)$ and generate $P^{(\text{fluc.})}(t_1)$ as described above using $\sigma_{MB}^{(1)}$, $\mu_{MB}^{(1)}$ and $\gamma_{MB}^{(1)}$. For the next interval we take different local parameters, i.e. for $t_2 \in (T, 2T)$ we apply $\sigma_{MB}^{(2)}$, $\mu_{MB}^{(2)}$ and $\gamma_{MB}^{(2)}$ and so on. The full trajectory is then given by combining the individual local trajectories, i.e. $P^{(\text{fluc.})}(t) = \left\{P^{(\text{fluc.})}(t_1), P^{(\text{fluc.})}(t_2), ..., P^{(\text{fluc.})}(t_M)\right\}$.

Considering $J \neq 3$

We consider the general stochastic model given by

$$P^{(\text{fluc.})}(t) = \sqrt{(x_1(t))^2 + ... + (x_J(t))^2} + \mu_{MB}, \tag{9}$$

$$\mathrm{d}x_i(t) = -\gamma_{MB} x_i(t)\,\mathrm{d}t + \sqrt{2\gamma_{MB}}\sigma_{MB}\mathrm{d}W_i, \tag{10}$$

where we now consider $J = 1...5$. To illustrate the dependency of the probability distribution on the number of random variables considered, we compare synthetic data with $J = 1, ..., 5$ with the local snapshots of power fluctuations from Fig. 6(b), (c) of the main text. These are one broad and one narrow distribution, approximately Maxwell-Boltzmann, based on NOVAREF data from April 2018. For all J, we determine the parameters γ_{MB} and σ_{MB} as before so that the combined $P^{(\mathrm{fluc.})}$ approximates the standard deviation of the empirical data. We display the results in Supplementary Fig. 16 and 17: For all J, the PDF has a similar shape but is increasingly shifted (Supplementary Fig. 16). We compute the log-likelihood that the synthetic data reproduces the real data and note that $J = 2$ and $J = 3$ are the most likely choices. Since the mathematical relations for $J = 3$ are well established from statistical physics, we use this.

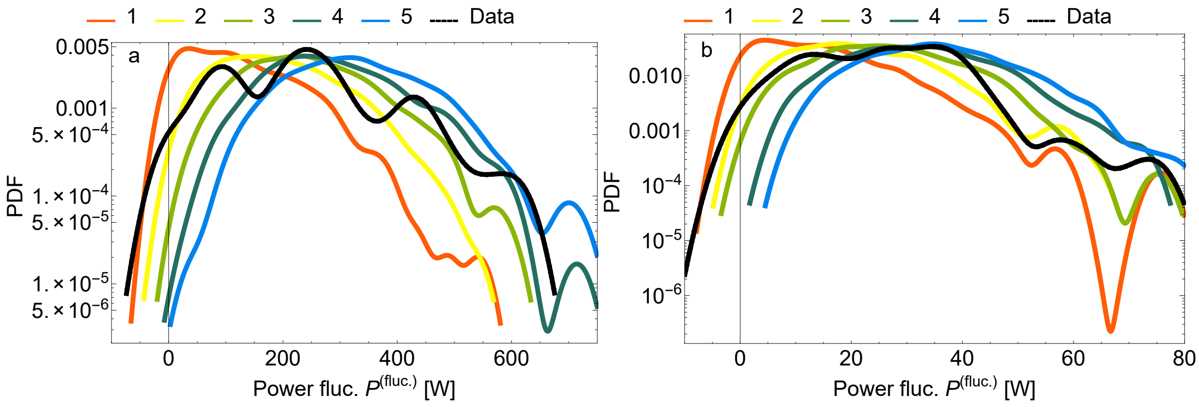

Supplementary Figure 16. **For $J = 1...5$, all processes are a decent fit to the empirical data** We compare the synthetic distributions generated using eq. (10) with one broad and one narrow distribution from the empirical data, based on the NOVAREF data from April. The more processes are included in the sum, the more the distribution shifts to the right and particularly $J \geq 4$ is no longer a great description of the data.

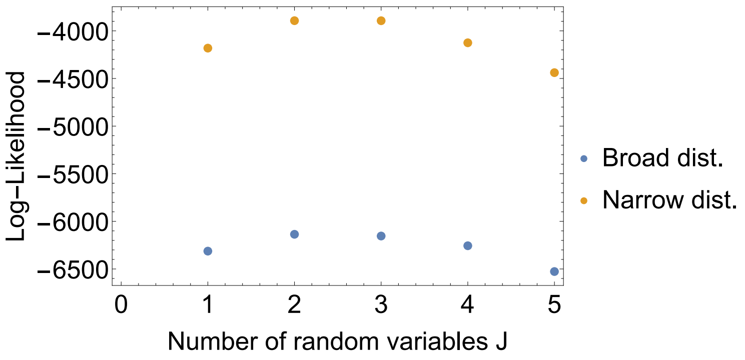

Supplementary Figure 17. **The most likely number of independent variables J is given between $J = 2$ and $J = 3$** We compute the likelihood for the different J values to fit the empirical data presented in Supplementary Fig. 16 where "broad" and "narrow" distributions refer to the two different snapshots from the NOVAREF data from April, see also main text Fig.6.

Compare real and synthetic data

Our synthetic model is indeed capable of reproducing the observed statistics. We consider the following cases: i) Real data from the NOVAREF data set for the time period 01.04-08.04, ii) our synthetic model with the local Maxwell-Boltzmann distributions, characterised by its mean and scale parameters μ_{MB} and σ_{MB}, respectively generated in the order they appear in the historic time series and iii) our synthetic model but with a μ_{MB} and σ_{MB} randomly drawn from the values obtained from the historic time series (see Supplementary Figs. 18 and 19). The real ordering works such that $\sigma_{MB}^{\mathrm{Synt}}(t) = \sigma_{MB}^{\mathrm{Real}}(t)$ for $t \in (0, T)$ and the same for $t \in (T, 2T)$ and so, i.e. for each time interval T, we determine the best fitting scale parameter σ_{MB}, shift μ_{MB} and autocorrelation decay γ and use those values in the same interval, i.e. the same time stamps when generating the synthetic trajectory. In contrast, for the random case, we only draw scale parameter σ_{MB}, shift μ_{MB} from the observed distributions, see Supplementary Fig. 15 and set the autocorrelation decay as the mean of the distribution from Supplementary Fig. 14.

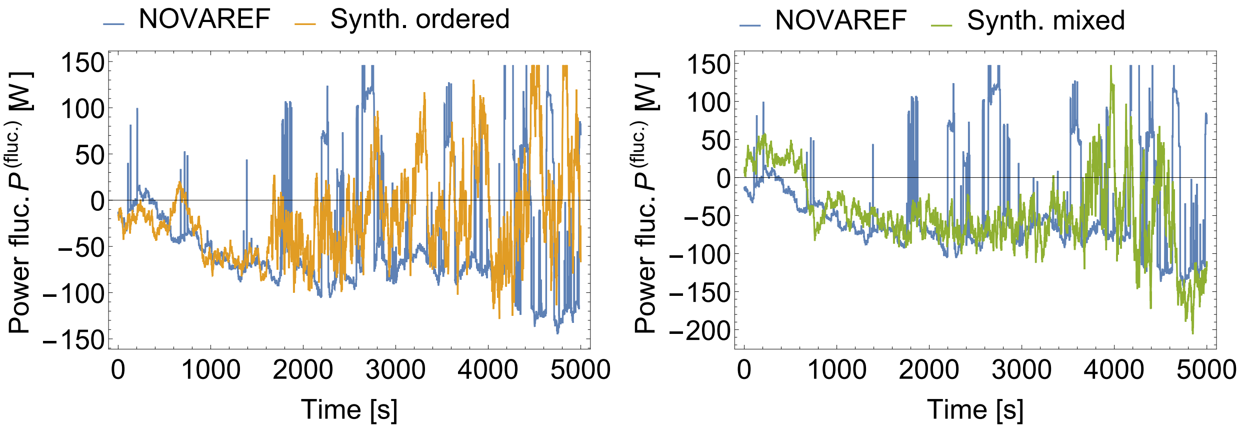

Supplementary Figure 18. **The real data and the synthetic Maxwell-Boltzmann process generates similarly looking trajectories** Left: We compare synthetic data generated by applying μ and σ values in the same order as they appear in the real data with real data from April of the NOVAREF data set. Right: We use the same real data but this time the synthetic data is generated by randomly choosing μ and σ, based on their respective distributions from April of the NOVAREF data set. Randomly choosing the distribution parameters leads to no close agreement of the trajectories, while the overall aggregated statistics is very similar, see Supplementary Fig. 19.

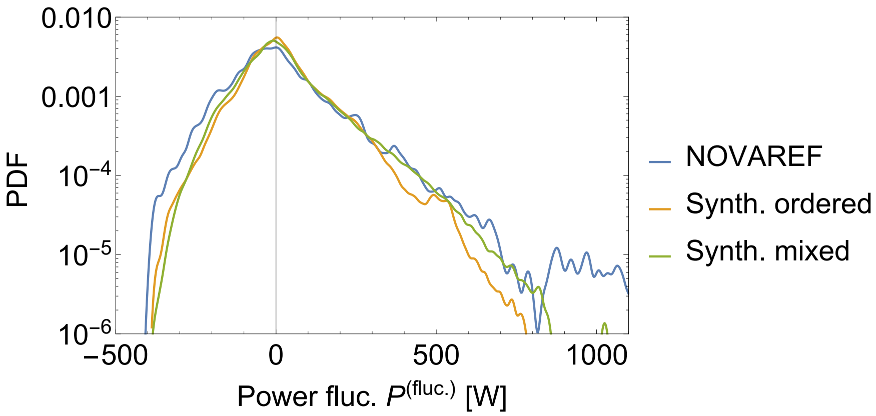

Supplementary Figure 19. **The histogram of the real data and the synthetic Maxwell-Boltzmann process** The histograms, i.e. the aggregated probability distributions to observe a certain variation in consumption, are very similar for the real data, the synthetic data using the real ordering and the randomised (mixed) synthetic data. The real data is drawn from early April of the NOVAREF data set.

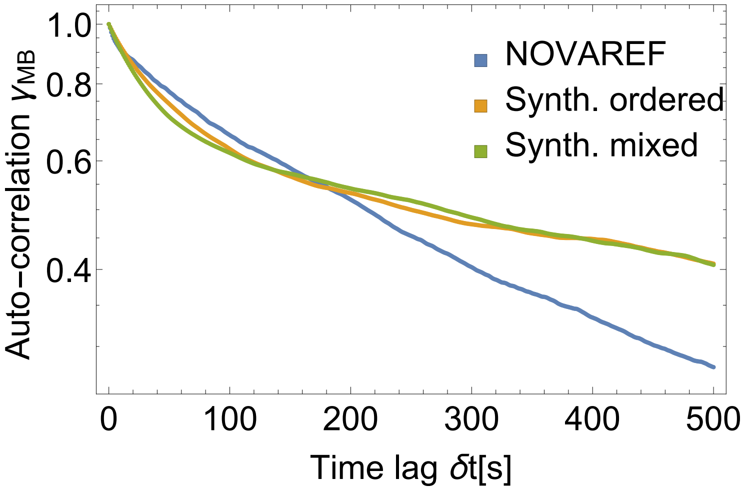

Supplementary Figure 20. **For the first 400 seconds, the autocorrelation of the synthetic and the real data agree very well** Here, we compare the synthetic data generated either using their historic ordering (ordered) or by randomly choosing μ and σ (mixed), based on their respective distributions in week 1 of April of the NOVAREF data set. The decay constants γ of the exponential decay $\exp(-\gamma\delta t)$ are $\gamma_{NOVAREF} \approx 0.0031/s$ and $\gamma_{Synth.} \approx 0.0025/s$.

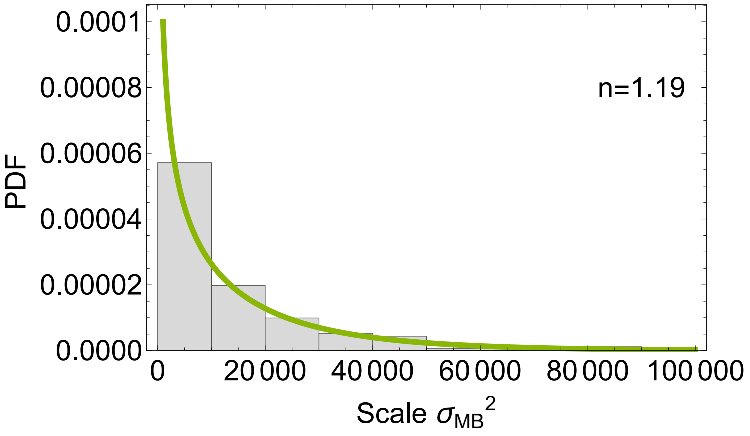

Supplementary Figure 21. χ^2 **distribution of** σ^2_{MB} The distribution of the squared scale parameters σ^2_{MB} follows approximately a χ^2 distribution for the NOVAREF data set.

χ^2 distributed σ^2_{MB}

We estimated the scale parameters of the local Maxwell-Boltzmann distributions as σ_{MB}. According to superstatistics theory, the exponential tails observed in the aggregated statistics could be explained by these scale parameters following a χ^2 distribution:

$$f(\beta) = \frac{1}{\Gamma\left(\frac{n}{2}\right)} \left(\frac{n}{2\beta_0}\right)^{\frac{n}{2}} \beta^{\frac{n}{2}-1} exp\left(-\frac{n\beta}{2\beta_0}\right), \tag{11}$$

where we set $\beta = \sigma^2_{MB}$ and β_0 as the mean of σ^2_{MB}.

Indeed, comparing this prediction with the σ^2_{MB} distributions of the NOVAREF data, we notice a very good agreement, see Supplementary Fig. 21.

Dependence of fluctuation results on the number of modes

In the main text we discussed the separation of the total demand into trend and fluctuations:

$$P = P^{\text{trend}} + P^{\text{fluc.}}. \tag{12}$$

We performed the split using Empirical Mode Decomposition (EMD). The precise number of modes attributed to either trend or fluctuation is not a given but we determined it using a minimisation of the normalized mean-squared error (NMSE). However, the minimum might not always be well-defined or different error measures might lead to a different number of optimal modes. Here, we demonstrate that while the quantitative results are changed, the qualitative results of the demand fluctuation analysis are unchanged when the number of modes is altered. For illustration purposes, we will consider three different fluctuation cases: using the 9, 10 or 11 highest-frequency modes of the 17 extracted modes and the residual signal, see Supplementary Figs. 22-27.

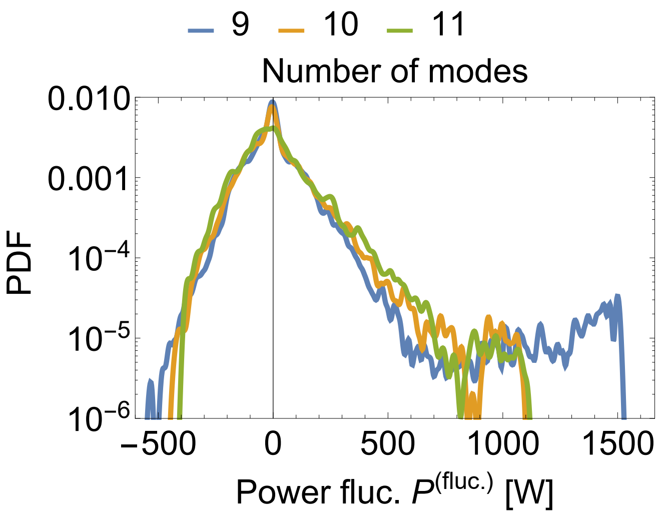

Supplementary Figure 22. **The probability density function (PDF) of the demand fluctuations does not change substantially with the number of modes** We include a varying number of modes to represent the fluctuations and the PDF stays very similar. Only the peak at fluctuations around zero vanishes when including the 11th mode.

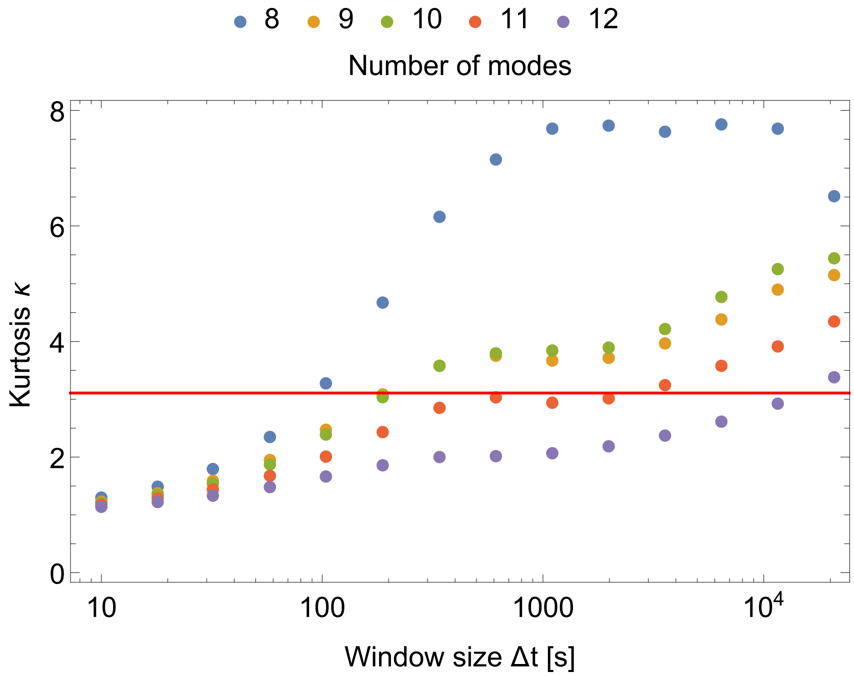

Supplementary Figure 23. **The kurtosis as a function of the time interval Δt decreases with increasing number of modes** We plot the local kurtosis for the time period Δt for a varying number of modes to represent the fluctuations. The red line is the kurtosis value of the Maxwell-Boltzmann distribution.

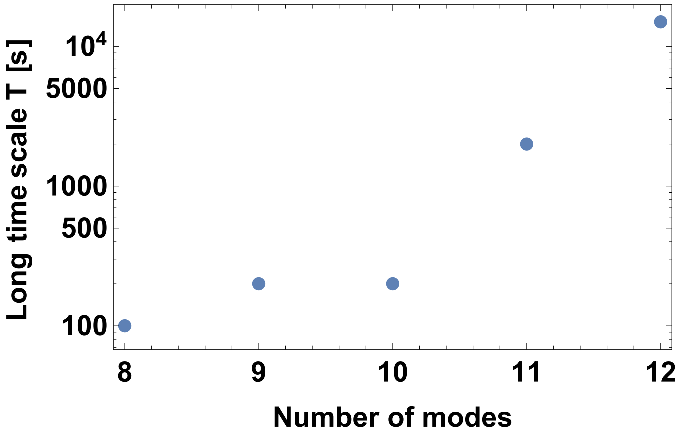

Supplementary Figure 24. **The long time scale increases with increasing number of modes** We plot the long time scale T determined by $\kappa(T) = \kappa(MB)$, i.e. the local kurtosis is equal to the Maxwell-Boltzmann kurtosis. This long time scale increases almost exponentially (noting the log-scale of the y-axis).

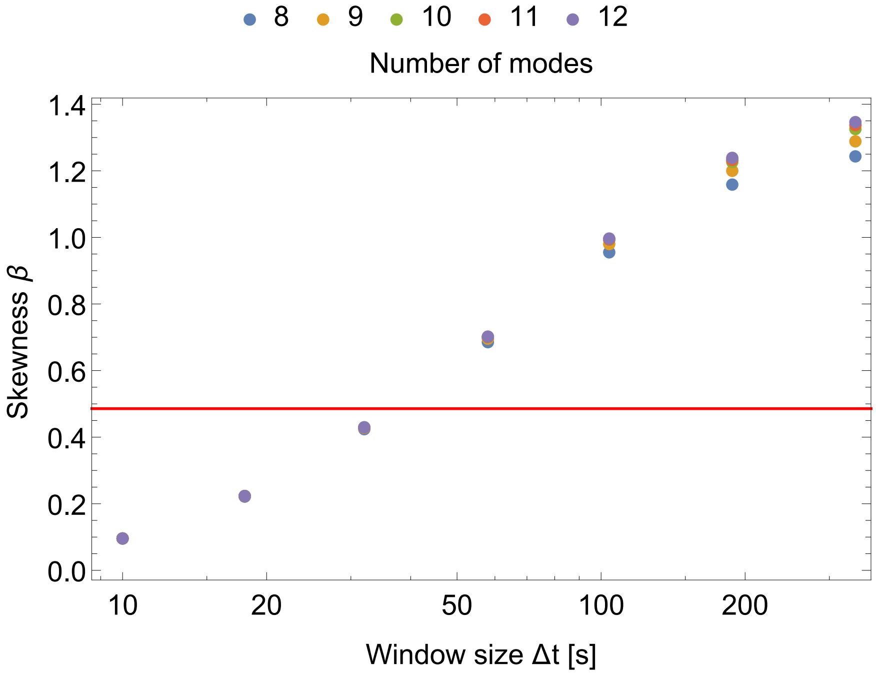

Supplementary Figure 25. **The local skewness is independent of the number of modes** We plot the local skewness for the time period Δt for a varying number of modes to represent the fluctuations. The red line is the skewness value of the Maxwell-Boltzmann distribution.

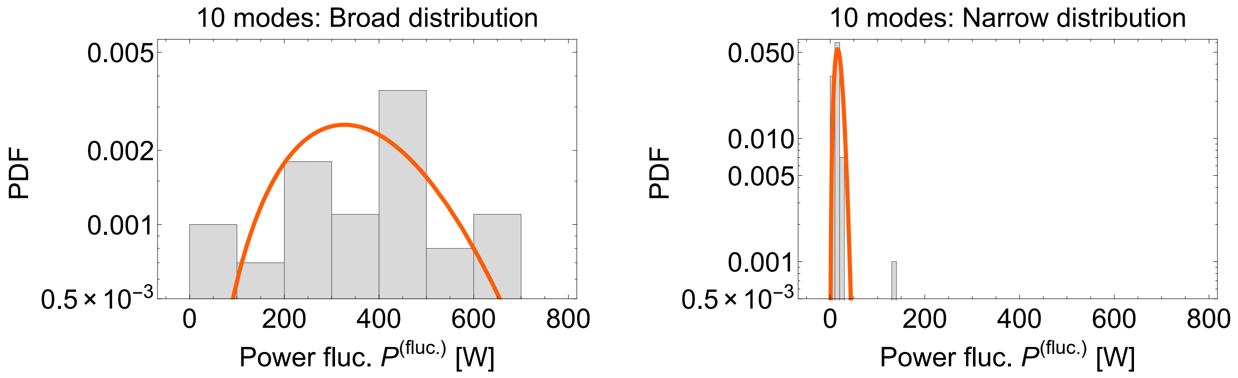

Supplementary Figure 26. **For 10 modes the 100 data points approximate a Maxwell-Boltzmann distribution** We plot two local Maxwell-Boltzmann distributions: A broad one, i.e one with large scale parameter σ_{MB} (Left) and a narrow one, i.e one with small scale parameter σ_{MB} (Right). The red lines give the best Maxwell-Boltzmann fit based on maximum likelihood.

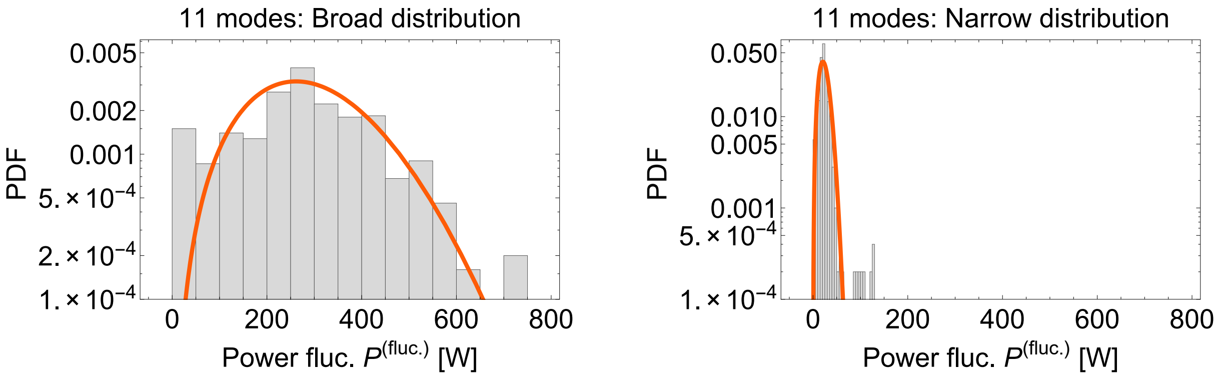

Supplementary Figure 27. **For 11 modes the 1000 data points approximate a Maxwell-Boltzmann distribution well** We plot two local Maxwell-Boltzmann distributions: A broad one, i.e one with large scale parameter σ_{MB} (Left) and a narrow one, i.e one with small scale parameter σ_{MB} (Right). The red lines give the best Maxwell-Boltzmann fit based on maximum likelihood.

Supplementary Note 5

Proof-of-concept predictions

Here we provide a proof-of concept of three different settings: First, how would our ALP change when a different error metric is considered? Secondly, can we train data in the summer and apply the ALP to data in the winter? Lastly, does the ALP procedure also work when applied to a different data set?

Let us first consider the usage of a different error metric for the same process used in Section *Demand trend: Mode decomposition*. In the main text, we determined the optimal number of modes via the mean-squared error (MSE), i.e. $\text{Mean}((P_{\text{ALP}} - P_{\text{measurement}})^2)$, where P_{ALP} is the averaged load profile obtained from the EMD analysis and $P_{\text{measurement}}$ is the measured electricity consumption obtained from averaging the recorded electricity consumption over all houses. Instead of the MSE, we could apply the mean-absolute error (MAE) defined as $Mean(|P_{\text{ALP}} - P_{\text{measurement}}|)$, where $|...|$ denotes the absolute value, and then consider $MAE_{fraction}$ as follows:

$$MAE_{fraction} = \frac{MAE_{ALP}}{MAE_{H0SLP}}. \tag{13}$$

If the $MAE_{fraction} < 1$, then the ALP can indicate the trend of electricity consumption of a group of houses more accurately than the H0 SLP can. Effectively, we move from an L2 loss function to an L1 loss function. As we can see in Supplementary Figures 28c and 29c, our ALP approach still works well and the overall shape of the error curve does not change drastically. Depending on the specific case, the optimal number of modes might change. In Table IV, we report also the values of MAE for twelve test weeks in winter time and compare them with corresponding H0 SLP ones. For this purpose, we reduce first the validation set to one week and shift these weeks to test set. As explained in Supplementary Note 3 to extract ALP from NOVAREF data set, one week validation set is enough. As seen in Table IV, MAE values of ALP are still less than H0 SLP ones, except one week (04.15-04.22). This can be expected as MAE can depend on the specific weeks and unexpected events may lead to an increased MAE.

Supplementary Table IV. **MAE values of the NOVAREF dataset for the winter time** The value of the MAE for the twelve weeks test set and its comparison with the H0 ALP MAE. By decreasing the validation set to one week and shifting these weeks to the test set, we calculate the MAE for each week. As can be seen, except for one week (04.15-04.22), the MAE value obtained from the ALP is less than the one from the H0 SLP.

Week	MAE ALP	MAE H0 SLP
03.11 - 03.18	101	106
04.01 - 04.08	105	109
04.08 - 04.15	106	113
04.15 - 04.22	110	108
04.22 - 04.29	115	120
05.06 - 05.13	118	127
05.13 - 05.20	114	123
05.20 - 05.27	109	120
05.27 - 06.03	107	117
06.03 - 06.10	111	121
06.10 - 06.17	103	116
06.17 - 06.24	107	114

In the main text, we train, validate and test the ALP approach without addressing seasonal effects. Here, we investigate how the ALP can still capture the trend of the power demand in winter more accurately than the H0 SLP, when only being trained in the summer. The process followed is the same as the one used in Section *Demand trend: Mode decomposition*. The "summer" NOVAREF data set (July-December) is split into training and validation sets and, then, winter weeks (January-June) are used as the test set.

As seen in Supplementary Figure 28b, c and d, the ALP still performs well in this case. Note that we extracted the modes by the EMD for the entire data set, including winter and summer. In Supplementary Fig. 28a part of EMD modes belonging to one day in summer is shown. One would expect that the ALP precision drops, if the EMD were only applied on days in one season. However, the results from the IDEAL data set (presented below), where we run the training on individual days, demonstrates very positive results. Further, we note that regions, such as deserts,

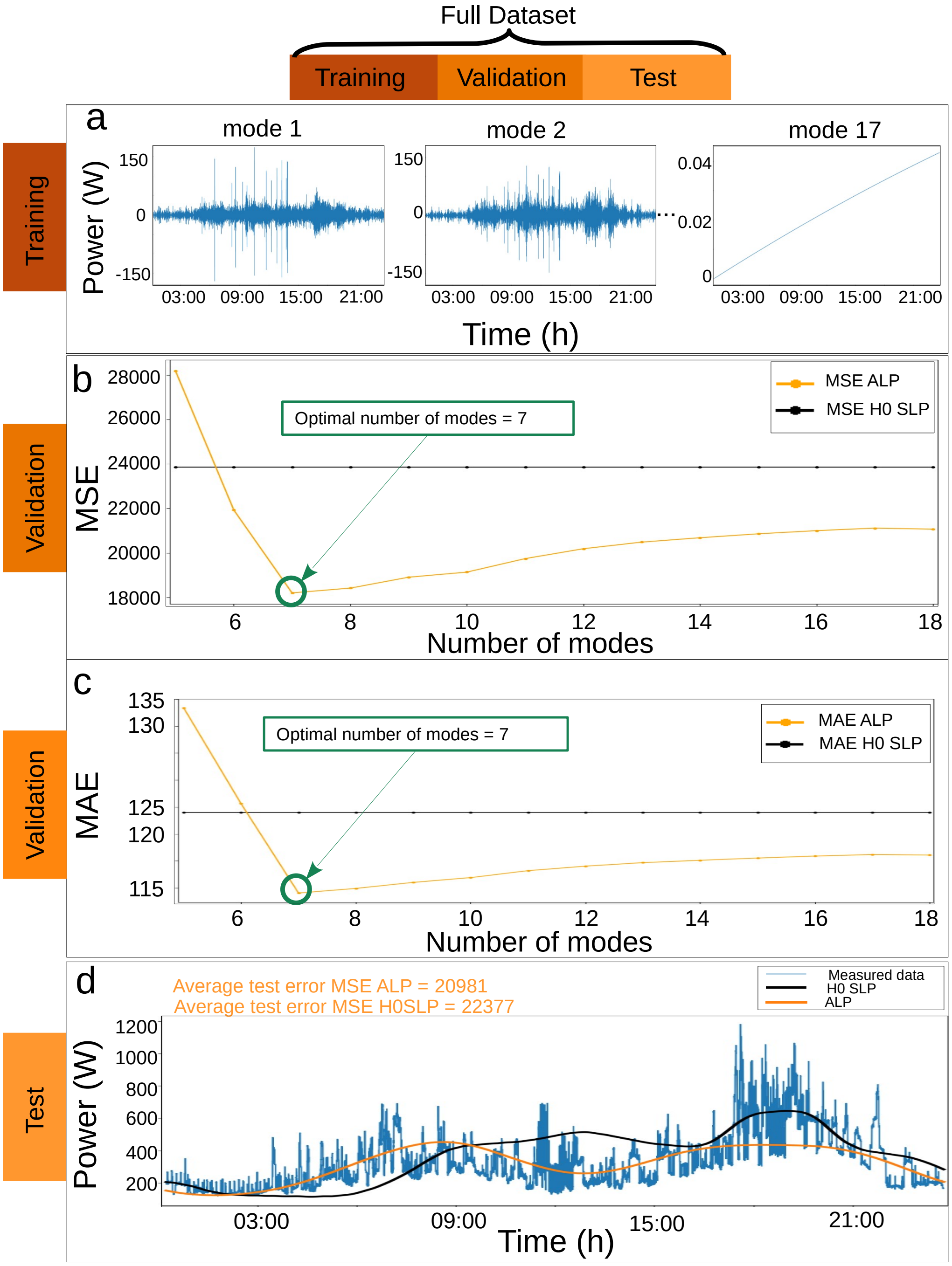

Supplementary Figure 28. **Training in the summer season and capturing the trend of the demand in the winter season works.** Similar to figure 3 in the main text, the full data set is split into training, validation and test sets (shown at the top of the Figure). (a) The individual modes of the summer time training set are trained to extract the $N_{optimal}$ from the validation set of the same season in (b) and, then, apply the optimum ALP on the winter weeks in (c), as the test set. (b)-(c) The error metric as a function of the number of modes calculated for the validation set using the mean-squared error (MSE) and the mean-absolute error (MAE). Both the MSE and MAE indicate that the ALP performs better than the H0 SLP for the majority of N values, because the MSE and MAE of the ALP are lower than those for H0 SLP. Moreover, both the MSE and MAE show that $N_{optimal} = 7$. (d) The optimal ALP model for the test set that outperforms the H0 SLP, as evident from its lower average value of MSE reported in the text above the graph. Here we evaluate the performance on data measured during winter (12.16-12.23) while training the model on data measured during the summer (July). The day depicted here is December 21st.

might show much more pronounced seasonal differences in their load profiles [61], potentially limiting the usage of

ALP without prior training on all seasons.

So far we have mainly demonstrated that the ALP works while using the NOVAREF data set. Here, we repeat a similar procedure as outlined in the main text (Section *Demand trend: Mode decomposition*) on the IDEAL data set. Due to the fact that the IDEAL data set has many gaps and, thus, it was impossible to extract enough weeks for the further analysis (see Supplementary Note 3 for further information), we changed our approach slightly. Instead of first applying the EMD on the full data set, we apply it separately on each day in the training set and, then, use modes obtained to train the ALP.

As we can see in Supplemental Figure 29, panels b, c and d, the ALP also outperforms the H0 SLP on the IDEAL data set for several different ALPs (each ALP created from a different set of mode sums). The optimal set of modes is reached here for a smaller number of modes compared to the NOVAREF case. Another interesting observation is that the MSE (and the MAE) of the IDEAL ALP is substantially below the H0 SLP baseline. Compared to the week-based analysis of the NOVAREF we can make the supposition that when shorter time frames are used to extract the EMD modes more short-time specific and less long-time averaged information (seasonal effects) are included.

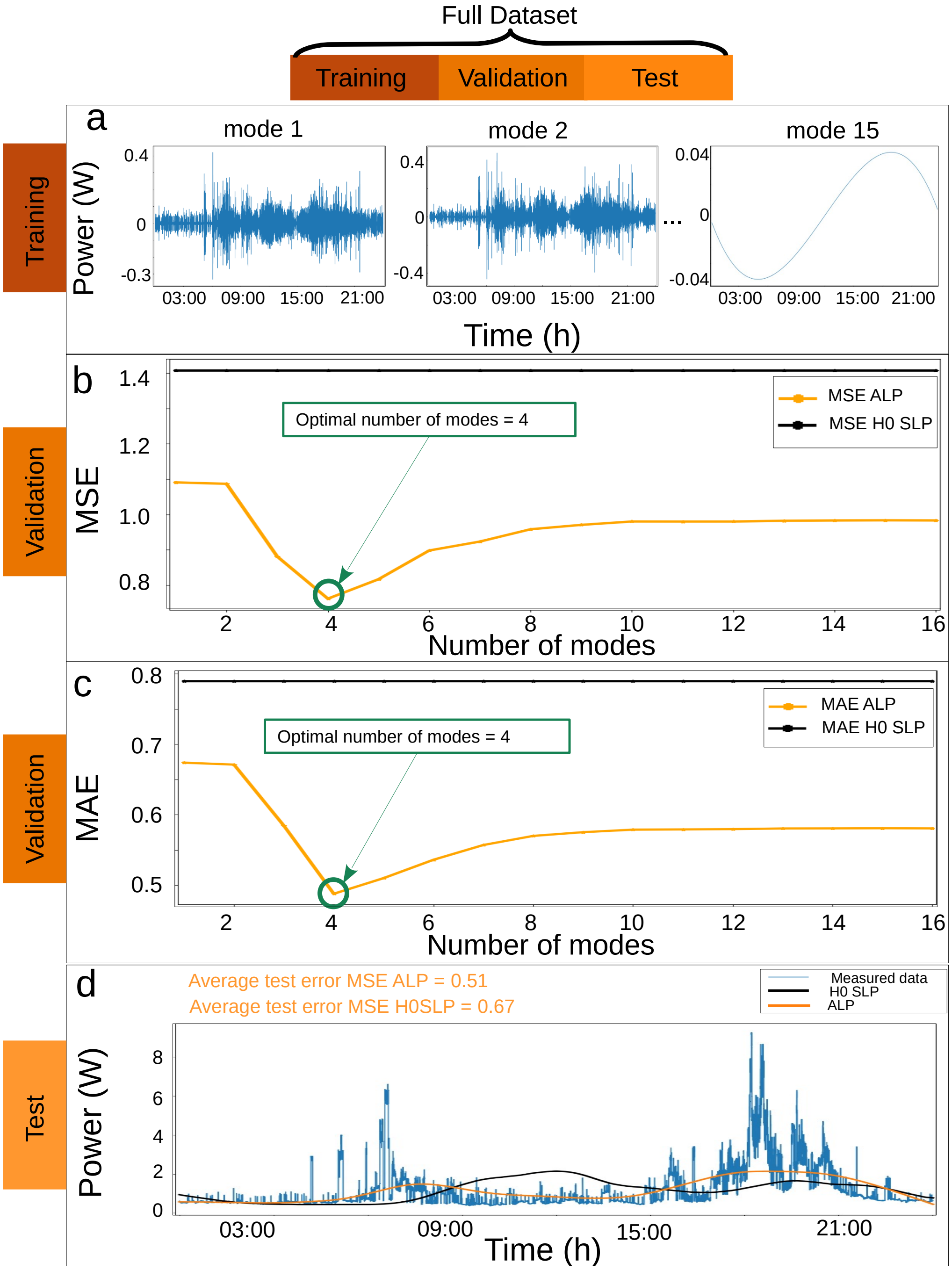

Supplementary Figure 29. **The ALP outperforms the H0 SLP for the IDEAL data set.** The full data set is split into training, validation and test sets (shown at the top of the Figure). Similar to Figure 3 of the main text, we plot (a) the individual modes which are used to train the ALP, (b)-(c) the error metric as a function of the number of modes, using the mean-squared error (MSE) and the mean-absolute error (MAE). Both the MSE and the MAE of the ALP for the validation set are substantially below the H0 SLP baseline, and both indicate that $N_{optimal} = 4$. (d) The optimal ALP model for the test set, which performs better than the H0 SLP, as evident from its lower average value of MSE reported in the text above the graph. Here, we evaluate the performance on data measured during a single day of the IDEAL data set, while training the model on data measured during several individual days from the same data set. The day depicted here is 26.03.2018.

Supplementary Note 6

Translation of German texts

The references 2 and 3 in the main text ([29, 57]) are unfortunately only available in German as these concern German and Austrian regulations.
To ease access, we provide both the German (original) and English (translated via *Google translate*) version on the OSF repository of this article: `https://osf.io/yu2dm/`

Supplementary References

---

[1] Proedrou, E. A comprehensive review of residential electricity load profile models. *IEEE Access* **9**, 12114–12133 (2021).

[2] Bartels, R., Fiebig, D. G., Garben, M. & Lumsdaine, R. An end-use electricity load simulation model: Delmod. *Utilities Policy* **2**, 71 – 82 (1992).

[3] Yao, R. & Steemers, K. A method of formulating energy load profile for domestic buildings in the uk. *Energy and Buildings* **37**, 663 – 671 (2005).

[4] Paatero, J. V. & Lund, D. A model for generating household electricity load profiles. *International Journal of Energy Research* **30**, 273–290 (2006). URL `https://onlinelibrary.wiley.com/doi/abs/10.1002/er.1136`.

[5] Ren, Z., Paevere, P. & McNamara, C. A local-community-level, physically-based model of end-use energy consumption by australian housing stock. *Energy Policy* **49**, 586 – 596 (2012). Special Section: Fuel Poverty Comes of Age: Commemorating 21 Years of Research and Policy.

[6] Shao, S., Pipattanasomporn, M. & Rahman, S. Development of physical-based demand response-enabled residential load models. *IEEE Transactions on Power Systems* **28**, 607–614 (2013).

[7] Gottwalt, S., Ketter, W., Block, C., Collins, J. & Weinhardt, C. Demand side managementa simulation of household behavior under variable prices. *Energy Policy* **39**, 8163 – 8174 (2011). URL `http://www.sciencedirect.com/science/article/pii/S0301421511008007`. Clean Cooking Fuels and Technologies in Developing Economies.

[8] Capasso, A., Grattieri, W., Lamedica, R. & Prudenzi, A. A bottom-up approach to residential load modeling. *IEEE Transactions on Power Systems* **9**, 957–964 (1994).

[9] Alzate, E. B., Mallick, N. H. & Xie, J. A high-resolution smart home power demand model and future impact on load profile in germany. In *2014 IEEE International Conference on Power and Energy (PECon)*, 53–58 (2014).

[10] Collin, A. J., Tsagarakis, G., Kiprakis, A. E. & McLaughlin, S. Development of low-voltage load models for the residential load sector. *IEEE Transactions on Power Systems* **29**, 2180–2188 (2014).

[11] Gao, B., Liu, X. & Zhu, Z. A bottom-up model for household load profile based on the consumption behavior of residents. *Energies* **11**, 2112 (2018). URL `http://dx.doi.org/10.3390/en11082112`.

[12] Armstrong, M. M., Swinton, M. C., Ribberink, H., Beausoleil-Morrison, I. & Millette, J. Synthetically derived profiles for representing occupant-driven electric loads in canadian housing. *Journal of Building Performance Simulation* **2**, 15–30 (2009). URL `https://doi.org/10.1080/19401490802706653`.

[13] Gruber, J. K. & Prodanovic, M. Residential energy load profile generation using a probabilistic approach. In *2012 Sixth UKSim/AMSS European Symposium on Computer Modeling and Simulation*, 317–322 (2012).

[14] Bajada, J., Fox, M. & Long, D. Load modelling and simulation of household electricity consumption for the evaluation of demand-side management strategies. In *IEEE PES ISGT Europe 2013*, 1–5 (2013).

[15] Marszal-Pomianowska, A., Heiselberg, P. & Larsen, O. K. Household electricity demand profiles a high-resolution load model to facilitate modelling of energy flexible buildings. *Energy* **103**, 487 – 501 (2016).

[16] McKenna, E. & Thomson, M. High-resolution stochastic integrated thermal–electrical domestic demand model. *Applied Energy* **165**, 445–461 (2016).

[17] McKenna, E., Thomson, M. & Barton, J. Crest demand model (2019).

[18] Neu, O., Sherlock, S. B., Oxizidis, S., Flynn, D. & Finn, D. Developing building archetypes for electrical load shifting assessment: Analysis of irish residential stock. Tech. Rep., Science Foundation Ireland (2014).

[19] Dickert, J. & Schegner, P. Residential load models for network planning purposes. In *2010 Modern Electric Power Systems*, 1–6 (2011).

[20] Fischer, D., Härtl, A. & Wille-Haussmann, B. Model for electric load profiles with high time resolution for german households. *Energy and Buildings* **92**, 170 – 179 (2015). URL `http://www.sciencedirect.com/science/article/pii/S0378778815000845`.

[21] Smart Energy Research Lab. Smart energy research lab exploratory data, 2019-2020: Secure access. [data collection]. 2nd edition. uk data service (2020). URL `http://doi.org/10.5255/UKDA-SN-8643-2`.

[22] Commercial and residential hourly load profiles for all TMY3 locations in the united states (2019). URL `https://openei.org/datasets/files/961/pub/ARCHIVE/`.

[23] Makonin, S. Hue: The hourly usage of energy dataset for buildings in British Columbia. Tech. Rep. (2019).

[24] AUSGRID Solar home electricity data (2011). URL `https://www.ausgrid.com.au/Industry/Our-Research/Data-to-share/Solar-home-electricity-data`.

[25] CER Smart Metering Project - Electricity Customer Behaviour Trial 2009-2010 [dataset], 1st edition. URL `https://www.ucd.ie/issda/data/commissionforenergyregulationcer/`.

[26] Tindemans, S. *et al.* Low carbon london project: Data from the dynamic time-of-use electricity pricing trial, 2013 (2016). URL `http://doi.org/10.5255/UKDA-SN-7857-2`.

[27] AECOM Building Engineering. Energy demand research project: Early smart meter trials, 2007-2010. [data collection] (2018). URL `http://doi.org/10.5255/UKDA-SN-7591-1`.

[28] EAM-Netz GmbH. Standardlastprofilverfahren (2019). URL `https://www.eam-netz.de/fuer-partner/netzzugang-und-netznutzung/strom/standardlastprofilverfahren/`.

[29] Bitterer, R. & Prof. Dr. habil. B. Schieferdecker. Repräsentative VDEW-Lastprofile Aktionsplan Wettbewerb, M-32/99. Tech. Rep., VDEW, Stresemannallee 23 D-60596 Frankfurt /M (2001).

[30] Bitterer, R. & Prof. Dr. habil. B. Schieferdecker. Praxistest Moderne Energiesparsysteme im Haushalt. Tech. Rep., IZES GmbH Institute für ZukunftsEnergie-und Stoffstromsysteme (2012). URL `http://www.izes.de/de/projekte/praxistest-moderne-energiesparsysteme-im-haushalt`.

[31] IEE PES-ISS Open Datasets (2020). URL `https://site.ieee.org/pes-iss/data-sets/`.

[32] Barker, S. *et al.* Smart*: An open data set and tools for enabling research in sustainable homes. *SustKDD, August* **111**, 108 (2012).

[33] Hebrail, G. & Berard, A. Individual household electric power consumption data set (2012). URL `bit.ly/1byLbr0`.

[34] Pereira, L., Quintal, F., Goncalves, R. & Nunes, N. J. Sustdata: A public dataset for ICT4S electric energy research. In *ICT for Sustainability 2014 (ICT4S-14)* (Atlantis Press, 2014). URL `https://doi.org/10.2991/ict4s-14.2014.44`.

[35] Saldanha, N. & Beausoleil-Morrison, I. Measured end-use electric load profiles for 12 canadian houses at high temporal resolution. *Energy and Buildings* **49**, 519–530 (2012). URL `https://carleton.ca/sbes/publications/electric-demand-profiles-downloadable/`.

[36] Batra, N., Gulati, M., Singh, A. & Srivastava, M. B. It's different: Insights into home energy consumption in india. In *Proceedings of the 5th ACM Workshop on Embedded Systems For Energy-Efficient Buildings*, 1–8 (2013).

[37] Makonin, S., Ellert, B., Bajić, I. V. & Popowich, F. Electricity, water, and natural gas consumption of a residential house in canada from 2012 to 2014. *Scientific data* **3**, 1–12 (2016).

[38] Johnson, G. & Beausoleil-Morrison, I. Electrical-end-use data from 23 houses sampled each minute for simulating micro-generation systems. *Applied Thermal Engineering* **114**, 1449–1456 (2017). URL `https://carleton.ca/sbes/publications/electric-demand-profiles-downloadable/`.

[39] Kolter, J. Z. & Johnson, M. J. Redd: A public data set for energy disaggregation research. In *In proceedings of the SustKDD workshop on Data Mining Applications in Sustainability* (2014).

[40] Murray, D. & Stankovic, L. Refit: Electrical load measurements (2015).

[41] The tracebase data set (2020). URL `https://github.com/areinhardt/tracebase`.

[42] Beckel, C., Kleiminger, W., Cicchetti, R., Staake, T. & Santini, S. The eco data set and the performance of non-intrusive load monitoring algorithms. In *Proceedings of the 1st ACM Conference on Embedded Systems for Energy-Efficient Buildings*, BuildSys '14, 8089 (Association for Computing Machinery, New York, NY, USA, 2014). URL `https://doi.org/10.1145/2674061.2674064`.

[43] Monacchi, A., Egarter, D., Elmenreich, W., D'Alessandro, S. & Tonello, A. M. Greend: An energy consumption dataset of households in Italy and Austria. In *2014 IEEE International Conference on Smart Grid Communications (SmartGridComm)*, 511–516 (IEEE, 2014).

[44] Einfalt, A. *et al.* ENERGIE DER ZUKUNFT Publizierbarer Endberich, ADRES-Concept. Tech. Rep., TU Wien (2012). URL `https://www.ea.tuwien.ac.at/fileadmin/t/ea/projekte/ADRES_Concept/PublizierbarerEndberichtADRES_815674.pdf`.

[45] Uttama Nambi, A. S., Reyes Lua, A. & Prasad, V. R. Loced: Location-aware energy disaggregation framework. In *Proceedings of the 2nd acm international conference on embedded systems for energy-efficient built environments*, 45–54 (2015).

[46] Makonin, S., Wang, Z. J. & Tumpach, C. Rae: The rainforest automation energy dataset for smart grid meter data analysis. *data* **3**, 8 (2018).

[47] Kelly, J. & Knottenbelt, W. The UK-DALE dataset, domestic appliance-level electricity demand and whole-house demand from five UK homes. *Scientific Data* **2** (2015).

[48] Anderson, K., Ocneanu, A., Carlson, D. R., Rowe, A. & Bergés, M. Blued: A fully labeled public dataset for event-based non-intrusive load monitoring research. In *BLUED: A Fully Labeled Public Dataset for Event-Based Non-Intrusive Load Monitoring Research* (2012).

[49] Shin, C. *et al.* The ENERTALK dataset, 15 Hz electricity consumption data from 22 houses in Korea. *Scientific data* **6**, 1–13 (2019).

[50] Anvari, M. *et al.* Data-driven load profiles and the dynamics of residential electric powerconsumption. *arXiv preprint arXiv:2009.09287* (2020).

[51] Goddard, N. *et al.* Ideal household energy dataset, 2016-2018 [dataset].

[52] Lange, M. & Zobel, M. NOVAREF, Erstellung neuer Referenzlastprofile zur Auslegung, Dimensionierung und Wirtschaftlichkeitsberechnung von Hausenergieversorgungssystemen. Tech. Rep., NEXT—ENERGY, Carl-von-Ossietzky-Strasse 15, 26129 Oldenburg (2016).

[53] enera: Der nächste grosse Schritt der Energiewende (2019). URL `https://projekt-enera.de/`.

[54] Pullinger, M. *et al.* The ideal household energy dataset, electricity, gas, contextual sensor data and survey data for 255 uk homes. *Scientific Data* **8**, 1–18 (2021).

[55] Ideal household energy dataset (2021). URL `https://datashare.ed.ac.uk/handle/10283/3647?show=full`.

[56] Fünfgeld, C. & Teidemann, R. Anwedung der Repräsentative VDEW-Lastprofile step-by-step, M-05/2000. Tech. Rep., VDEW, Stresemannallee 23 D-60596 Frankfurt /M (2001).

[57] E-Control. Sonstige MarktregelnStrom Kapitel 6 Zählwerte, Datenformate undstandardisierte Lastprofile. Tech. Rep., Energie-Control Austria für die Regulierung der Elektrizitäts- und Erdgaswirtschaft, Rudolfspl. 13A, 1010 Wien, Austria (2019). URL `https://www.e-control.at/recht/marktregeln/sonstige-marktregeln-strom`.

[58] Muratori, M. Impact of uncoordinated plug-in electric vehicle charging on residential power demand. *Nature Energy* **3**, 193–201 (2018).

[59] Hilshey, A. D., Hines, P. D. & Dowds, J. R. Estimating the acceleration of transformer aging due to electric vehicle charging. In *2011 IEEE Power and Energy Society General Meeting*, 1–9 (IEEE, 2011).

[60] Richardson, I., Thomson, M., Infield, D. & Clifford, C. Domestic electricity use: A high-resolution energy demand model. *Energy and Buildings* **42**, 1878–1887 (2010).

[61] Alrawi, O., Bayram, I. S., Al-Ghamdi, S. G. & Koc, M. High-resolution household load profiling and evaluation of rooftop pv systems in selected houses in qatar. *Energies* **12**, 3876 (2019).
